# PINK1-Dependent Mitophagy Reduced Endothelial Hyperpermeability and Cell Migration Capacity Under Simulated Microgravity

**DOI:** 10.3389/fcell.2022.896014

**Published:** 2022-07-07

**Authors:** Chengfei Li, Yikai Pan, Yingjun Tan, Yongchun Wang, Xiqing Sun

**Affiliations:** ^1^ Department of Aerospace Medical Training, School of Aerospace Medicine, Fourth Military Medical University, Xi’an, China; ^2^ China Astronaut Research and Training Center, Beijing, China

**Keywords:** simulated microgravity, endothelial cells, mitophagy, inflammasome, cell migration

## Abstract

The effect of cardiovascular dysfunction including orthostatic intolerance and disability on physical exercise is one of the health problems induced by long-term spaceflight astronauts face. As an important part of vascular structure, the vascular endothelium, uniquely sensitive to mechanical force, plays a pivotal role in coordinating vascular functions. Our study found that simulated microgravity induced PINK1-dependent mitophagy in human umbilical vein endothelial cells (HUVECs). Here, we explored the underlying mechanism of mitophagy induction. The ER stress induced by proteostasis failure in HUVECs promoted the Ca^2+^ transfer from ER to mitochondria, resulting in mitochondria Ca^2+^ overload, decreased mitochondrial membrane potential, mitochondria fission, and accumulation of Parkin and p62 in mitochondria and mitophagy under simulated microgravity. Moreover, we assumed that mitophagy played a vital role in functional changes in endothelial cells under simulated microgravity. Using mdivi-1 and PINK1 knockdown, we found that NLRP3 inflammasome activation was enhanced after mitophagy was inhibited. The NLRP3 inflammasome contributed to endothelial hyperpermeability and cellular migration by releasing IL-1β. Thus, mitophagy inhibited cell migration ability and hyperpermeability in HUVECs exposed to clinostat-simulated microgravity. Collectively, we here clarify the mechanism of mitophagy induction by simulated microgravity *in vitro* and demonstrate the relationship between mitophagy and vascular endothelial functional changes including cellular migration and permeability. This study deepens the understanding of vascular functional changes under microgravity.

## Introduction

Microgravity is a new stressor that no terrestrial organisms on Earth have ever encountered evolutionarily. The spaceflight environment presents a significant hazard to the musculoskeletal, pulmonary, immune, and cardiovascular systems of astronauts. Cardiovascular deconditioning is one of the major concerns due to changes in gravity. The endothelium, which is sensitive to mechanical stress, could control vasomotor responses by producing vasoactive signaling molecules such as VEGF, NO, and ET-1 and maintain the integrity of the vascular system by serving as the barrier. The endothelium as a mechano-transducer converts mechanical stimuli into biochemical signals, mediating vascular remodeling. Thus, gravitational unloading as a mechanical stimulus causes alteration of cell morphology and endothelial function. Exposure to simulated microgravity conditions causes a reduction in F-actin and β-tubulin expression (Janmaleki et al., 2016), sparse and discontinuous filaments, mitochondria vacuolization, and chromatin condensation and margination. Simulated microgravity increases the proportion of apoptotic choroidal vascular endothelial cells and decreases the amount of F-actin. However, different types of endothelial cells exhibit different functional changes concerning proliferation and migration under microgravity. For example, cell migration is promoted in HUVECs and impaired in porcine aortic endothelial cells exposed to microgravity. The proliferation rate is decreased in murine microvascular endothelial cells, whereas the proliferation is inhibited in HUVECs under simulated microgravity (Maier et al., 2015; Zhao et al., 2020). Our previous studies have reported that autophagy in HUVECs induced by clinostat-simulated microgravity facilitates cellular migration and inhibits apoptosis by mitigating ER stress (Li et al., 2018a; Li et al., 2019). However, there are few studies on the cytoplasmic organelle damage induced by microgravity in endothelial cells.

Mitochondria are energy-producing powerhouses of the cell, which are exquisitely sensitive to stress-related damage. In recent years, more light has been shed on mitochondrial dysfunction after the perception of hypogravity. A study including human cell models and astronaut metabolites reveals altered mitochondrial function such as imbalances in the assembly of OXPHOS complexes and mtDNA gene expression changes in many tissues, suggesting mitochondria as a critical hub for spaceflight impact on human biology (da Silveira et al., 2020). Similarly, genomic and functional changes in mitochondria such as mtRNA and mitochondria respiration after a 1-year spaceflight mission have been observed in astronauts (Garrett-Bakelman et al., 2019). Recent studies have indicated that microgravity alters mitochondria morphology in the A549 cell line, which is verified by dilated cristae with increased electron density (Degan et al., 2021). Moreover, some studies reveal the mtUPR in cardiomyocytes and nematodes under simulated microgravity (Feger et al., 2016; Liu et al., 2019). The damaged mitochondria, mitochondria vacuolization, and decline in mitochondrial content are found under simulated microgravity (Jeong et al., 2018; Zhao et al., 2020). Together, these studies suggest the possible degradative mechanism that disposes of damaged mitochondria. A recent study has found that disassembly of the cytoskeleton induces mitophagy, contributing to HUVEC adaption to gravitational unloading (Locatelli et al., 2020). Our previous study reveals that 2-D clinorotation induces autophagy in HUVECs through the HDM2-p53-mTOR pathway (Li et al., 2018a). Thus, we questioned whether clinorotation could trigger the elimination of damaged mitochondria through autophagy and the underlying molecular mechanism in HUVECs. However, which simulated microgravity leads to enhanced mitophagy remains elusive.

Although mitochondrial Ca^2+^ is required for activation of dehydrogenases in the Krebs cycle and ATP synthase, Ca^2+^ overload leads to mitochondrial fission, Ca^2+^ influx, mitochondria potential depolarization, mitophagy, and an increase in ROS production (Yang et al., 2019). The ER is the major calcium storage organelle in mammalian cells. Ca^2+^ flux between the ER and mitochondria is a key step in mediating cellular responses to stress conditions. ER stress is reported to enhance ER Ca^2+^ leak toward mitochondria (Chami et al., 2008; Koshenov et al., 2021). Our group previously reported that ER stress and UPR occur in HUVECs during microgravity exposure (Li et al., 2019). Whether ER stress under simulated microgravity could facilitate Ca^2+^ transfer from the ER to mitochondria and lead to mitochondrial Ca^2+^ overload remains to be investigated.

Studies have shown that microgravity activates inflammatory responses in several types of cells. Long-duration space microgravity causes amplified pro-inflammatory cytokine production in the immune system, triggering a hyperinflammatory phenotype (Buchheim et al., 2019). The gene expressions of IL-1, IL-6, and TNF involved in inflammation were found to be elevated in HUVECs in spaceflight (Versari et al., 2013). The NLRP3 inflammasome is the most fully characterized trigger-initiating inflammatory response so far. NLRP3, a cytosolic pattern recognition receptor, is activated in response to a broad range of pathogen-derived and endogenous agents. NLRP3 assembles an inflammasome complex with ASC to activate caspase-1, which drives the maturation and secretion of the pro-inflammatory cytokines, IL-1β and IL-18, and concurrently promotes the pathogenesis of inflammatory diseases. Many studies have indicated that the NLRP3 inflammasome links inflammatory stimuli to endothelial dysfunction. Trimethylamine-N-oxide induces activation of the NLRP3 inflammasome, leading to inflammasome-dependent endothelial hyperpermeability (Boini et al., 2017). Hyperglycemia-associated endothelial dysfunction and atherosclerotic lesions are related to the NLRP3 inflammasome in type 2 diabetes mellitus (Gora et al., 2021). A recent study has shown that clinostat-simulated microgravity induces NLRP3 inflammasome formation and activation in HUVECs, contributing to endothelial inflammation and apoptosis (Jiang et al., 2020). Given that the angiogenesis and cellular migration are shown to be changed after clinorotation in HUVECs, the role of the NLRP3 inflammasome in endothelial functions such as migration and permeability under microgravity is worthy of in-depth study.

It is of note that many studies have revealed that autophagic elimination of damaged mitochondria negatively regulates NLRP3 inflammasome activation. Quercetin alleviates NLRP3 inflammasome activation *via* promoting mitophagy in microglia (Han et al., 2021). ROS causes mitochondrial dysfunction, and mitophagy proved to alleviate the NLRP3 inflammasome by inhibiting ROS production (Chen et al., 2019; Lin et al., 2019). As far as we are aware, no information exists to know the role of mitophagy in NLRP3 inflammasome activation and endothelial dysfunction under microgravity conditions.

Keeping all the aforementioned findings in view, we are interested in elucidating the underlying molecular mechanisms of mitophagy and interplay between mitophagy, NLRP3 inflammasome, and endothelial dysfunction in HUVECs following clinostat-simulated microgravity exposure. We hypothesized that microgravity exposure induces mitophagy/mitochondrial dysfunction in HUVECs *via* ER stress, thereby alleviating endothelial dysfunction by inhibiting NLRP3 inflammasome activation. Thus, this study adds to the current understanding and support that mitophagy is one of the characteristics of simulated microgravity-associated endothelial organelle damage which plays an important role in endothelial dysfunction.

## Materials and Methods

### Cell Culture and Simulated Microgravity

The primary HUVEC cell line was purchased from the American Type Culture Collection (ATCC, Manassas, VA) and cultured in a cell incubator at a temperature of 37°C and a CO_2_ concentration of 5%. Microgravity conditions were simulated using a 2-D clinostat (Supplementary Figure S1). The direction changes through continuous rotation deprive the cells of the capacity to perceive gravity. Similar changes in the gene expression and cell morphology have been observed under clinorotation as compared to real microgravity (Herranz et al., 2013), suggesting the validity of this system for simulating the effects of microgravity. In this study, HUVECs were seeded on a 2.55 × 2.15 cm coverslip at a density of 1 × 10^5^ cells. Each coverslip was then placed in each well of 6-well plates. When the cells reached a confluence of 60%, the coverslips were placed into the fixture of the chambers (Astronaut Research and Training Center, Beijing, China), which were subsequently filled with a complete cell culture medium, removing air bubbles to avoid the effects of shear stress. Then, the chambers were rotated around the horizontal axis at a speed of 24 r/min before the cells were harvested (Supplementary Figure S1D). The control group was rotated around the vertical axis at the same speed (Supplementary Figure S1E).

### Special Reagents

Special reagents used in this study were purchased as indicated: mdivi-1(338967-87-6, TargetMol, Boston, Massachusetts, USA), MG132 (133407-82-6, Apexbio, Suzhou, China), NAC (616-91-1, Apexbio), human IL-1β neutralizing antibody (mabg-hil1b-3, InvivoGen, San Diego, CA, United State), MitoTEMPO (ab144644, Abcam, Cambridge, United Kingdom), and 2-APB (ab120124, Abcam).

### Mitochondrial Isolation

Cell mitochondrial extraction kits (C3601, Beyotime, Shanghai, China) were used for mitochondrial isolation of HUVECs according to the manufacturer’s instructions. The mitochondria and cytoplasm were isolated through differential centrifugation and kept in a storage solution with PMSF (1mM, ST506, and Beyotime) for Western blot analysis.

### TEM

The cells were digested and centrifuged after clinorotation for 48 h. Then, the cell precipitate was fixed in 2.5% glutaraldehyde for two hours at 4°C and post-fixed with osmic acid, dehydrated, embedded, and cut. After double staining with lead citrate and uranyl acetate, the images were acquired using a TEM system (HT7800, Hitachi, Japan).

### MitoTracker and LysoTracker

The cells on the coverslips after clinorotation were incubated in media containing 250 nmol/L MitoTracker Deep Red FM (40743ES50, Yeasen, Shanghai, China) for 30 min at 37°C and washed with pre-warmed PBS. The cells were then incubated in media containing 50 nmol/L LysoTracker Green DND-26(40738ES50, Yeasen) for 1 h in the cell incubator and observed under a confocal microscope (LSM 800, Zeiss, Oberkochen, Germany) to determine the red and green fluorescence.

### Transfection of siRNA and Overexpressing Plasmid

Knockdown of PINK1, MMP1, NLRP3, and HSF1 was performed by reverse transfection of PINK1 siRNA (GenePharma, Shanghai, China), MMP1 siRNA (GenePharma), NLRP3 siRNA (GenePharma), and HSF1 siRNA (GenePharma). RBM3-overexpression plasmid pGV141-RBM3 was purchased from GeneChem (Shanghai, China). The cells were placed into the chambers of a 2-D clinostat 24 h after being transfected with the abovementioned siRNA or plasmid by using Lipofectamine 2000 (11668-019, Invitrogen, Waltham, Massachusetts, United States).

### Western Blot

The protein from lysates of HUVECs was separated using 12% sodium dodecyl sulphate polyacrylamide gel electrophoresis and transferred onto PVDF membranes (IPVH00010, Millipore, Billerica, MA, United States). After blocking in 4% milk, the membranes were incubated with primary antibodies overnight at 4°C. The primary antibodies used are as follows: Tom20 (1:2000 dilution, 11802-1-AP, Proteintech, Rosemont, USA), Tim23 (1:1000 dilution, 11123-1-AP, Proteintech), PINK1(1:1000 dilution, ab137361, Abcam), Parkin (1:1000 dilution, 14060-1-AP, Proteintech), Drp1 (1:1000 dilution, 12957-1-AP, Proteintech), Mfn2 (1:1000 dilution, 12186-1-AP, Proteintech), p62 (1:1000 dilution, 18420-1-AP, Proteintech), caspase 1(1:1000 dilution, ab179515, Abcam), ZO-1 (1:1000 dilution, 20742-1-AP, Proteintech), occludin (1:1000 dilution, 13409-1-AP, Proteintech), NLRP3 (1:1000 dilution, 19771-1-AP, Proteintech), IL-1β (1:1000 dilution, ab2105, Abcam), MMP1 (1:1000 dilution, 10371-2-AP, Proteintech), RBM3 (1:1000 dilution, 14363-1-AP, Proteintech), Hsp70 (1:1000 dilution, 10995-1-AP, Proteintech), Hsp90 (1:5000 dilution, 60318-1-Ig, Proteintech), clusterin (1:1000 dilution, 12289-1-AP, Proteintech), COXIV (1:1000 dilution, 11242-1-AP, Proteintech), and GAPDH (1:2000 dilution, EK020, Zhuangzhi Biotech, Xi’an, China). The membranes were subsequently washed and incubated with HRP-conjugated secondary antibody (1:5000 dilution, EK020, Zhuangzhi Biotech) for 1 h at room temperature. The proteins were detected using an ECL Western blot detection reagent (WBKLS0500, Millipore). The gray value was quantified using ImageJ software (1.31v, Bethesda, USA).

### Immunofluorescence

After clinorotation, the cells growing in coverslips were fixed in 4% paraformaldehyde for 15 min at room temperature and rinsed several times with PBS. Then, the cells on coverslips were permeabilized in PBS with 0.1% Triton X‐100 (9036-19-5, Sigma-Aldrich, St. Louis, MO, United States) for 15 min and blocked with goat serum for 1 h. The coverslips were incubated in Tom20 (1:100 dilution, 11802-1-AP, Proteintech), LC3 (1:100 dilution, ab192890, Abcam), PINK1 (1:100 dilution), and Hsp60 (1:100 dilution, 15282-1-AP, Proteintech) primary antibodies in blocking solution at 4°C overnight. Fluor 488 (1:500 dilution, EK011, Zhuangzhi Biotech) or Cy3 (1:500 dilution, EK022, Zhuangzhi Biotech) secondary antibodies were used for incubation for 1 h in the dark at room temperature. The cells were then treated with DAPI solution (1:1000 dilution) for 5 min in the dark, washed with PBS, and kept at 4°C until imaged. The cells were visualized using laser confocal microscopy (LSM 800, Zeiss). The quantification of colocalization was performed using ImageJ software.

### qRT-PCR

RNA was extracted using TRIzol reagent (10296010, Thermo Fisher Scientific, Waltham, Massachusetts, United States), according to the manufacturer’s instructions. cDNA was generated using the PrimeScript II First-Strand cDNA Synthesis Kit (RR036A, Takara, Shiga, Japan) as described in the manufacturer’s protocol. Then, we performed quantitative PCR using SYBR Green reagent (RR420L, Takara) on a CFX96 Real‐Time System (Bio‐Rad, Hercules, CA). The sequences are listed in Table 1. The comparative CT method was used to calculate the relative amounts of mRNA.

**TABLE 1:** Primers used for amplification of the genes.

Name	Sequence (5′-3′)
*MMP1*-Forward	GAT​ATT​GGA​GCA​GCA​AGA​GG
*MMP1*-Reverse	CAC​CTT​CTT​TGG​ACT​CAC​AC
*GAPDH*- Forward	GTC​AAG​GCT​GAG​AAC​GGG​AA
*GAPDH*- Reverse	TCG​CCC​CAC​TTG​ATT​TTG​GA
*MMP2*-Forward	CAT​TCC​GCT​TCC​AGG​GCA​CAT
*MMP2*-Reverse	GCA​CCT​TCT​GAG​TTC​CCA​CCA​A
*MMP9*-Forward	TTT​GAC​AGC​GAC​AAG​AAG​TGG
*MMP9*-Reverse	TCC​CAT​CCT​TGA​ACA​AAT​ACA
*HSP70*-Forward	TGC​CCT​ATC​CAG​ATC​CTG​CTA
*HSP70*-Reverse	GAG​CCA​TCA​GAC​TGA​GGA​GTG​A
*HSP90*-Forward	TTCAGGCCCTTCCCGAAT
*HSP90*-Reverse	TCA​CTC​CTT​CCT​TGG​CAA​CAT
*CLU*-Forward	GAG​CAG​CTG​AAC​GAG​CAG​TTT
*CLU*-Reverse	CTTCGCCTTGCGTGAGGT
*HSP60*-Forward	CCG​AAG​ACG​TTG​ACG​GAG​AGG​C
*HSP60*-Reverse	TGA​CTG​CCA​CAA​CCT​GAA​GAC​CAA

### Proteasome Activity Assay

Proteasome activity was analyzed using a proteasome activity assay kit (ab107921, Abcam), which included fluorescent substrates that were Succ-LLVY-AMC specific for the chymotrypsin-like activity. The cell lysate was prepared using 0.5% NP-40 in dH_2_O. The supernatant was collected after centrifugation. Samples, standards, and positive control were seeded to a 96-well plate. The proteasome inhibitor MG132, positive control, and proteasome substrate were added to wells according to the manufacturer’s instructions and incubated for 10 min at 37°C. Fluorescence intensity was analyzed using a microplate reader (51119670DP, Thermo Fisher Scientific). The substrates were incubated for a further 30 min in the dark at 37°C and fluorescence intensity was analyzed again. The excitation and emission wavelengths were 350 and 440 nm, respectively. The standard curve was constructed, and the proteasome activity was calculated according to the manufacturer’s instructions.

### SUnSET Assay

SUnSET is a nonradioactive method to monitor protein synthesis (Schmidt et al., 2009). Briefly, the extent of puromycin incorporation into the nascent peptides is assessed using Western blot after the treatment with antibiotic puromycin, a tyrosyl-tRNA analog. The cells were removed from the wells of 6-well plates after clinorotation and incubated with fresh media containing puromycin (10 μg/ml, ab141453, Abcam) for 30 min. Subsequently, cellular protein lysates were obtained for Western blot analysis. The primary antibody was anti-puromycin (12D10, Millipore) at the dilution of 1:10000.

### JC-1 Staining

JC-1 dye formed aggregates inside the healthy mitochondrial matrix with red fluorescence while effluxes from the depolarized mitochondria to the cytoplasm acting as monomers with green fluorescence. After clinorotation, the cells on the coverslips were moved to wells of 6-well plates loaded with JC-1 dye (c2006, Beyotime) in the dark at 37°C for 20 min. The cells were washed and incubated in the assay buffer. The images were captured using laser confocal microscopy (LSM 800, Zeiss).

### Mitochondrial Ca^2+^ Detection

After clinorotation, the cells were washed with PBS and incubated in PBS containing 1 µM Rhod-2 AM (T19052, TargetMol) for 15 min at 37°C. Then, the cells were washed with PBS three times prior to incubation with PBS for 20 min at 37°C. The fluorescence images were captured using laser confocal microscopy (LSM 800, Zeiss). The excitation and emission wavelengths were 540 and 590 nm, respectively.

### ELISA

Human IL-1β in the cell culture medium was quantified using a human IL-1β ELISA kit (JL13662-48T, J&L Biological, Shanghai, China) according to the manufacturer’s instructions.

### Endothelial Permeability

HUVECs seeded on transwell inserts with a filter of 3-μm pore (3492, Corning, New York, United States) were exposed to clinorotation for 48 h. The transwell inserts were moved into 6-well plates with 1.5 ml fresh media. 100μL FITC–dextran (70kDa, 60842-46-8, Seebio, Shanghai, China) solution was added to each insert, and the plate was incubated at 37°C for 2 h to allow fluorescein molecules to flow through the endothelial cell monolayer. The media below the inserts were added to 96-well plates. The fluorescent intensity in each well was determined at excitation/emission of 485/530 nm using a microplate reader (51119670DP, Thermo Fisher Scientific). The arbitrary fluorescence intensity was used to calculate the relative permeability.

### Analysis of ROS and Mitochondrial ROS

Cellular ROS levels were detected using a DCFH-DA probe (ab113851, Abcam). Briefly, cells on the coverslips after clinorotation were washed with PBS three times before incubating with 5 μM DCFH-DA for 30 min at 37°C. After washing twice with a serum-free cell culture medium, we measured fluorescence using laser confocal microscopy (LSM 800, Zeiss). MitoSOX (40778ES50, Yeasen) was used to detect the mitochondrial ROS level in HUVECs. The cells were incubated in 5 μM MitoSOX for 30 min at 37°C, and positive staining was subsequently detected using fluorescence microscopy for imaging.

### Transwell Migration Assay

Transwell migration assay was performed using transwell inserts (3422, Corning) with a 8 μm pore filter. Cells exposed to clinorotation for 48 h were seeded into the upper chamber of the insert, and 600 μl of complete medium was added to the lower chamber. After 24 h incubation at 37°C, cells on the lower surface were fixed with methanol and stained with 0.2% crystal violet for 10 min. After washing with PBS three times, the cells were observed using an Olympus IX73 microscope.

### Wound Healing Assay

Briefly, the coverslips on which the HUVECs grew after clinorotation were removed into a 6-well plate and grown to nearly 100% confluence. Linear scratches were made on confluent cell monolayers using a sterile 200-μl pipette tip. The cells were then incubated with serum-free medium. Images were taken at 0 and 24 h with an Olympus IX73 microscope, and the rate of wound healing was measured using ImageJ software.

### Statistics

Data derived from three independent experiments were shown as mean ± standard deviation. SPSS software (version 19.0, IL, United States) was used for one-way ANOVA or Student’s t-test whenever appropriate. In all experiments, the differences were considered significant when *p* < 0.05.

## Results

### Clinostat-Simulated Microgravity-Induced Mitophagy *Via* the PINK1-Parkin Pathway in HUVECs

The mitochondria dynamic has been tied to mitochondrial quality control. Damaged or dysfunctional mitochondria undergo fragmentation from the healthy mitochondrial network. The fission of mitochondria is the requisite step for mitochondria to be accommodated within autophagosomes. Mitochondrial fission protein Drp1 and fusion protein Mfn1/Mfn2 mediate the structure alteration of mitochondria. We found that clinorotation for 24 and 48 h increased Drp1 protein expression and reduced the protein level of Mfn2 (Supplementary Figure S2A). To further investigate the altered mitochondrial dynamics, HUVECs were stained for the mitochondria marker Hsp60 before mitochondrial morphology was analyzed using confocal microscopy. As shown in Supplementary Figure S2B, confocal imaging revealed that mitochondria appeared fragmented after clinorotation for 48 h. Depolarization of mitochondria is known to induce their fragmentation (Legros et al., 2002). To determine whether mitochondrial depolarization occurred under clinorotation in HUVECs, the mitochondrial membrane potential in living cells was measured using the JC-1 probe and observed using confocal imaging. As shown in Supplementary Figure S2C, exposure to simulated microgravity for 48 h resulted in decreased membrane potential of mitochondria, which was reflected by the decreased ratio of red to green fluorescence.

**FIGURE 1:**
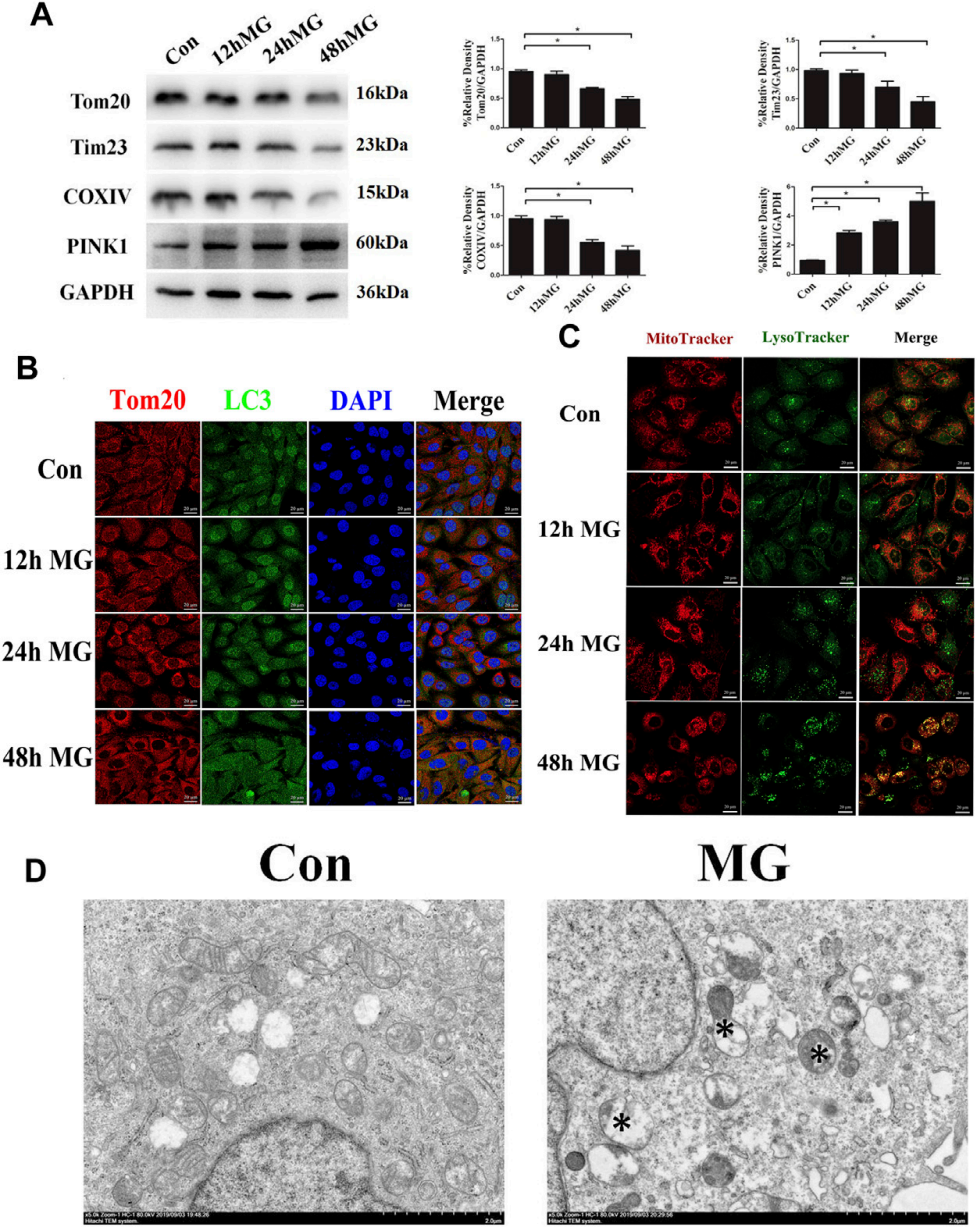
Mitophagy level was enhanced in HUVECs under clinorotation. HUVECs were exposed to clinostat-simulated microgravity for 12, 24, and 48 h. **(A)** Representative Western blot showing the expression of mitochondrial membrane proteins Tom20 and Tim23 as well as mitochondrial respiratory chain complexes COXIV. GAPDH was probed as a protein loading control. **(B)** Representative fluorescence images of cells showing the colocalization of LC3 (green) and Tom20 (red) through immunocytochemistry. **(C)** Representative fluorescence images showing the colocalization of mitochondria and lysosomes in HUVECs treated with MitoTracker (red) and LysoTracker (green) after exposure of cells to clinorotation. **(D)** Transmission electron microscopic images of mitochondrial ultrastructure and mitophagosomes (asterisk) in HUVECs after simulated microgravity exposure for 48 h. Data shown represented mean ± SD from triplicate experiments. *p < 0.05 vs. the control. Scale bar: 20 μm.

Next, we explored the effect of simulated microgravity on the initiation of mitophagy. Western blot confirmed that the mitochondrial proteins Tom20, Tim23, and COXIV were decreased after 24 and 48 h clinorotation (Figure 1A). Furthermore, exposure to clinorotation upregulated the expression of the mitophagy marker PINK1 at 12 h, with a sustained increase up to 48 h (Figure 1A). LC3 colocalized with Tom20 in HUVECs under clinorotation for 48 h, and the colocalization of MitoTracker and LysoTracker significantly increased in HUVECs under clinorotation for 48 h (Figures 1B,C). To further validate the mitophagy in HUVECs under clinorotation, we explored the formation of mitophagosomes using TEM and found increased mitophagosomes and phagolysosomes in HUVECs after clinorotation for 48 h (Figure 1D).

**FIGURE 2:**
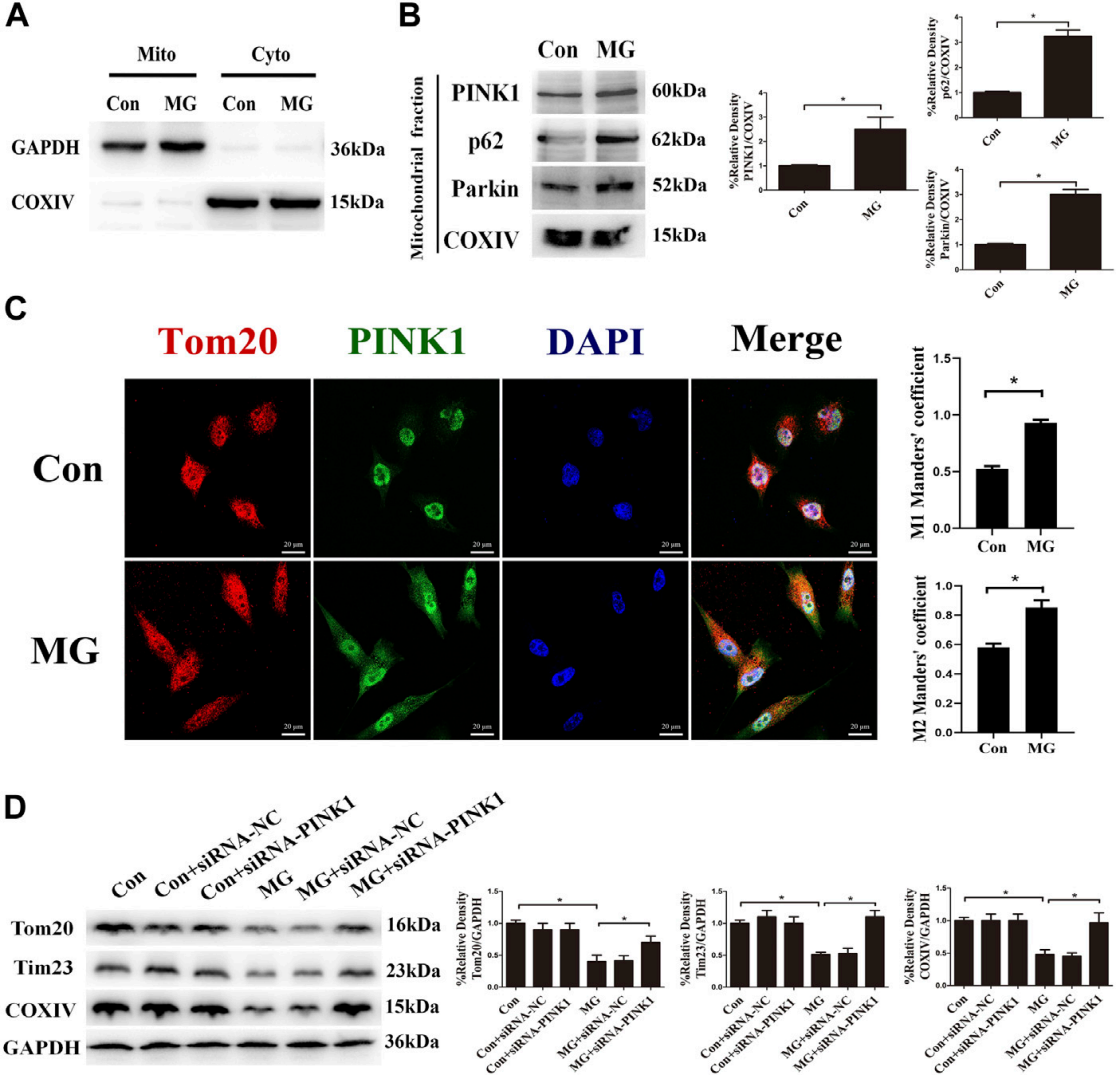
Clinorotation induced mitophagy through the activation of the PINK1/Parkin signaling pathway in HUVECs. **(A)** Cell lysates of HUVECs were divided into cytosolic fractions (Cyto) and mitochondrial fractions (Mito). Representative Western blot showing the expression of the cytosolic protein GAPDH and the marker protein of mitochondria COXIV to verify successful enrichment of mitochondrial fractions. **(B)** Representative Western blot showing the expression of PINK1, Parkin, and p62, proteins associated with mitophagy, in mitochondrial extracts in HUVECs exposed to microgravity for 48 h. COXIV was probed as a protein loading control for mitochondrial extracts. **(C)** Representative fluorescence images showing the colocalization of Tom20 (red) and PINK1 (green) in HUVECs exposed to clinorotation for 48 h. The Mann–Whitney U test was used for nonparametric data, and Manders’ coefficients M1 and M2 to quantify the colocalization. **(D)** Representative Western blot showing the expression of Tom20, Tim23, and COXIV in HUVECs transfected with either siRNA-NC or siRNA-PINK1 under clinorotation for 48 h. Data shown represented mean ± SD from triplicate experiments. *p < 0.05 vs. the control. Scale bar: 20 μm.

PINK1 is degraded in the healthy mitochondrial matrix. The depolarization of mitochondria stabilizes PINK1 on the outer membrane of mitochondria, leading to Parkin recruitment. The mitochondrial outer membrane proteins such as Mfn1 and Mfn2 are ubiquitination substrates of the cytosolic ubiquitin ligase Parkin. The polyubiquitinated cargo recruits p62, ultimately leading to autophagosome formation coupled with mitochondria *via* LC3-interacting domain. Given that the expression of PINK1 was increased in response to clinorotation, we hypothesized that clinorotation triggers PINK1/Parkin-dependent mitophagy. To examine PINK1, Parkin, and p62 accumulation on mitochondria in HUVECs under clinorotation for 48 h, we investigated their expression in mitochondrial extracts. We found that PINK1, Parkin, and p62 were recruited to mitochondria under clinorotation (Figure 2A), demonstrating that the PINK1/Parkin pathway was activated. As expected, PINK1 notably colocalized with Tom20 in HUVECs under clinorotation for 48 h (Figure 2B), thereby confirming the recruitment of PINK1 to mitochondria. To further confirm the role of PINK1 in the mitophagy induction process, the mitophagy level was evaluated in cells that were transfected with PINK1 siRNA and then exposed to clinorotation for 48 h. As expected, gene silencing of PINK1 inhibited the clinorotation-induced downregulation of mitochondrial proteins Tom20, Tim23, and COXIV (Figure 2C), thus underscoring the role of PINK1 in the induction of mitophagy in HUVECs under clinorotation.

### ER Stress Triggered Mitochondrial Fission and Mitophagy *via* Ca^2+^ Transfer Through IP_3_R in HUVECs Under Simulated Microgravity

**FIGURE 3:**
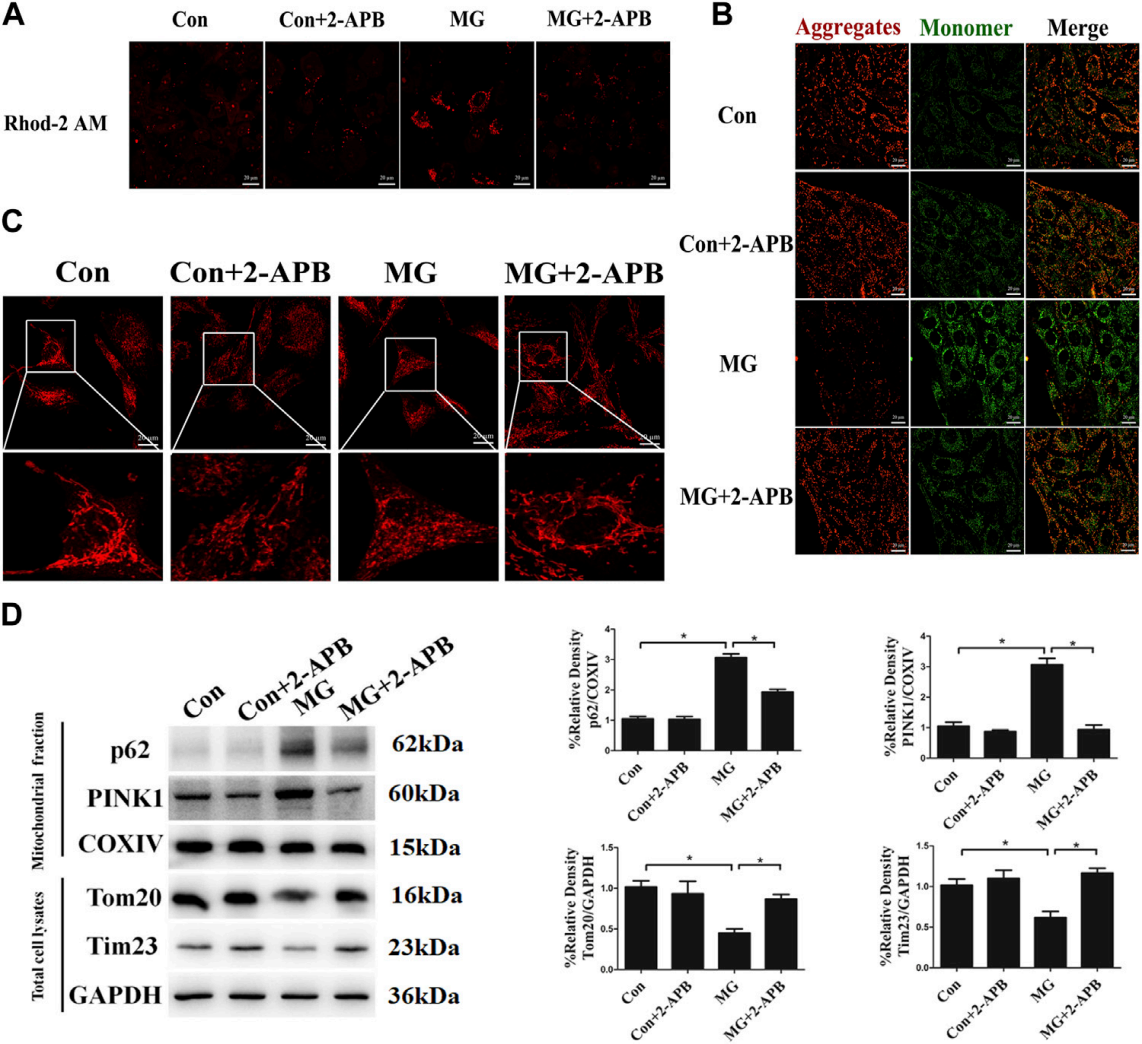
Clinorotation induced mitophagy by ER–mitochondria Ca^2+^ Transfer through IP_3_R in HUVECs. **(A)** Representative fluorescence images of HUVECs exposed to clinorotation for 48 h with or without 2-APB (10 μM), indicating mitochondrial Ca^2+^ levels using Rhod-2 AM, a mitochondrion-specific chemical Ca^2+^ indicator. **(B)** JC-1 assay was used to examine mitochondrial membrane potential in HUVECs under clinorotation for 48 h with or without 2-APB (10 μM). **(C)** Immunofluorescence staining for Hsp60 (red) to indicate the morphology of mitochondria in HUVECs exposed to clinorotation for 48 h with or without 2-APB (10 μM). **(D)** Representative Western blot showing the expression of PINK1, p62, and COXIV in mitochondrial extracts and Tom20 and Tim23 in total cell lysates of HUVECs exposed to clinorotation for 48 h with or without 2-APB (10 μM). COXIV was probed as a protein loading control for mitochondrial extracts. GAPDH was probed as a protein loading control for total cell lysates. Data shown represented mean ± SD from triplicate experiments. *p < 0.05 vs. the control. Scale bar: 20 μm.

We have discovered that microgravity largely affected mitochondrial dynamics and the level of mitophagy. However, a detailed mechanism through which clinorotation induces mitophagy remains unclear. The ER has a great influence on mitochondrial morphology and function. Alterations in ER–mitochondria crosstalk and ER stress are reported to be closely related to the impairment of mitochondrial function, fission, and mitophagy (Wang et al., 2021). Ca^2+^ transfer is the vital crosstalk between the ER and mitochondria. To explore the influence of ER–mitochondria Ca^2+^ transfer on the level of mitophagy, we detected the level of Ca^2+^ in the mitochondria after clinorotation using the Rhod-2 AM probe, a mitochondrion-specific chemical Ca^2+^ indicator. The fluorescence signal of Rhod-2 AM rose after clinorotation, and the presence of 2-APB, which is classically used to inhibit IP_3_R, dramatically decreased mitochondrial Ca^2+^ levels upon stimulation with simulated microgravity (Figure 3A), supposedly by blocking Ca^2+^ transfer and mitochondrial Ca^2+^ uptake.

It is reported that mitochondria exposed to Ca^2+^ overload exhibit reduced mitochondrial membrane potential, increased fission, and ROS production (Buhlman et al., 2014; Park et al., 2017). Less functional mitochondria activate the elimination of defective mitochondria by mitophagy. We determined whether excessive mitochondrial Ca^2+^ accumulation impacted the morphology, the membrane potential of mitochondria, and the level of mitophagy under clinorotation. As shown in Figures 3B and C, 2-APB ameliorated the decreased mitochondrial membrane potential and the fission of mitochondria in HUVECs under simulated microgravity. Next, we showed that 2-APB reversed the increased protein levels of p62, PINK1, and COXIV in mitochondrial fraction and the decreased protein level of Tom20 and Tim23 in the cell lysate under clinorotation (Figure 3D). These findings confirmed that ER–mitochondria Ca^2+^ transfer through the IP_3_R channel is the leading cause of mitophagy induction.

**FIGURE 4:**
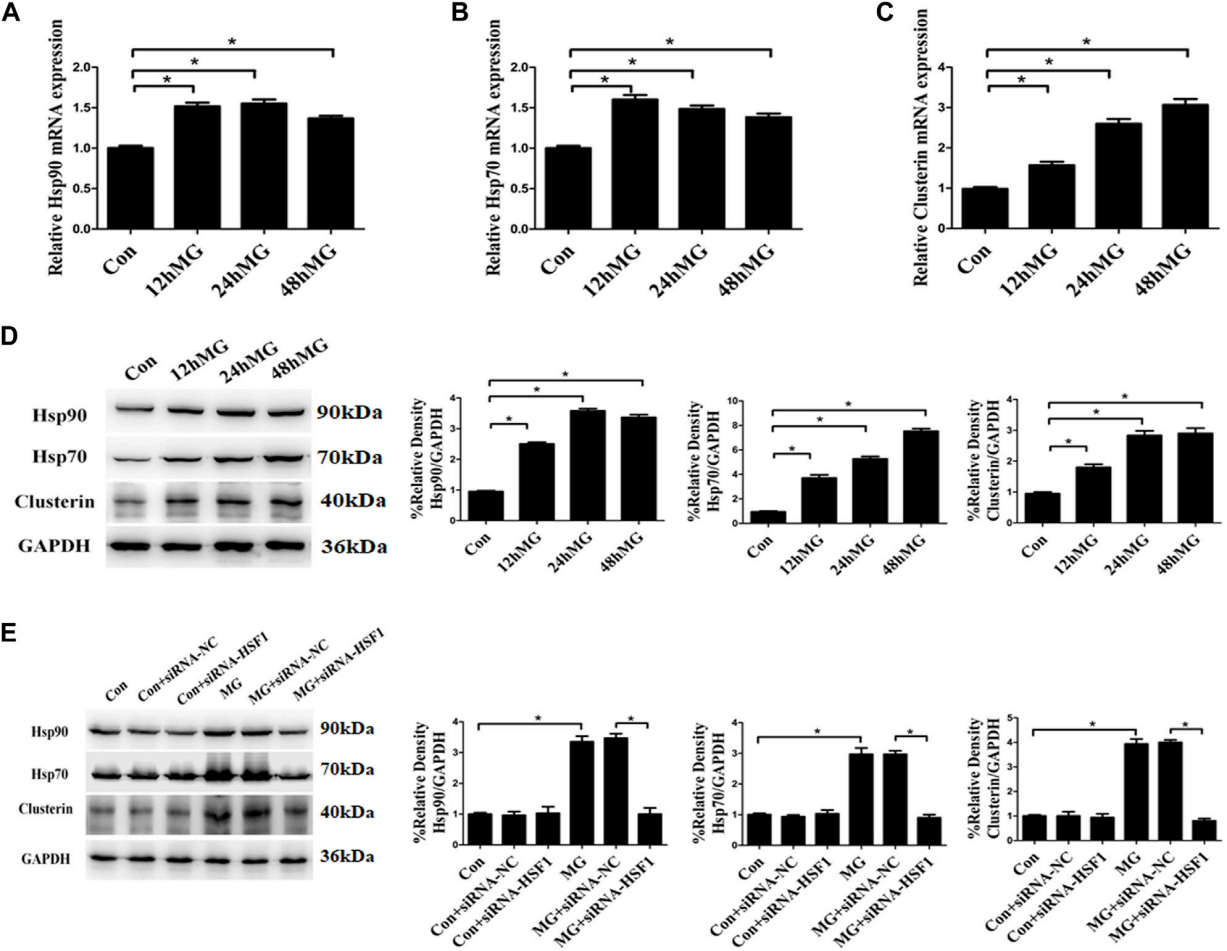
Clinostat-simulated microgravity induced heat shock response in HUVECs. **(A,B,C)** mRNA expression of heat shock proteins Hsp90 **(A)**, Hsp70 **(B),** and clusterin **(C)** in HUVECs under clinorotation for 12, 24, and 48 h. **(D)** Representative Western blot showing the expression of heat shock proteins Hsp90, Hsp70, and clusterin in HUVECs under clinorotation for 12, 24, and 48 h. **(E)** Representative Western blot showing the expression of Hsp90, Hsp70, and clusterin in HUVECs transfected with siRNA-NC or siRNA-HSF1 under clinorotation for 48 h. Data shown represented mean ± SD from triplicate experiments. *p < 0.05 vs. the indicated group.

Cellular stress, especially ER stress, has been shown to modulate the interaction between the ER and mitochondria, thus causing Ca^2+^ in ER to flow to mitochondria (Bravo et al., 2011). Our previous study has confirmed that clinorotation induces ER stress and UPR (Li et al., 2019). As the protein aggregate has been shown to induce ER stress and influence the ER–mitochondria Ca^2+^ crosstalk (Hedskog et al., 2013), we analyzed the impact of simulated microgravity on proteostasis in HUVECs. The accumulation of unfolding proteins due to cytoplasmic proteotoxic stimuli triggers transcriptional activation of HSF1, which promotes the expression of genes encoding chaperone proteins such as Hsp70, Hsp90, and clusterin. Chaperone proteins maintain protein homeostasis by stabilizing protein structures and assisting in the correct folding and unfolding of proteins (Neef et al., 2011). Exposure to clinorotation for 12, 24, and 48 h increased the expression of Hsp70, Hsp90, and clusterin at both mRNA and protein levels in HUVECs (Figures 4A–D). The effect of clinorotation on the expression of Hsp70, Hsp90, and clusterin was abolished by HSF1 knockdown (Figure 4E). The heat shock response suggested proteotoxic stress under clinorotation.

**FIGURE 5:**
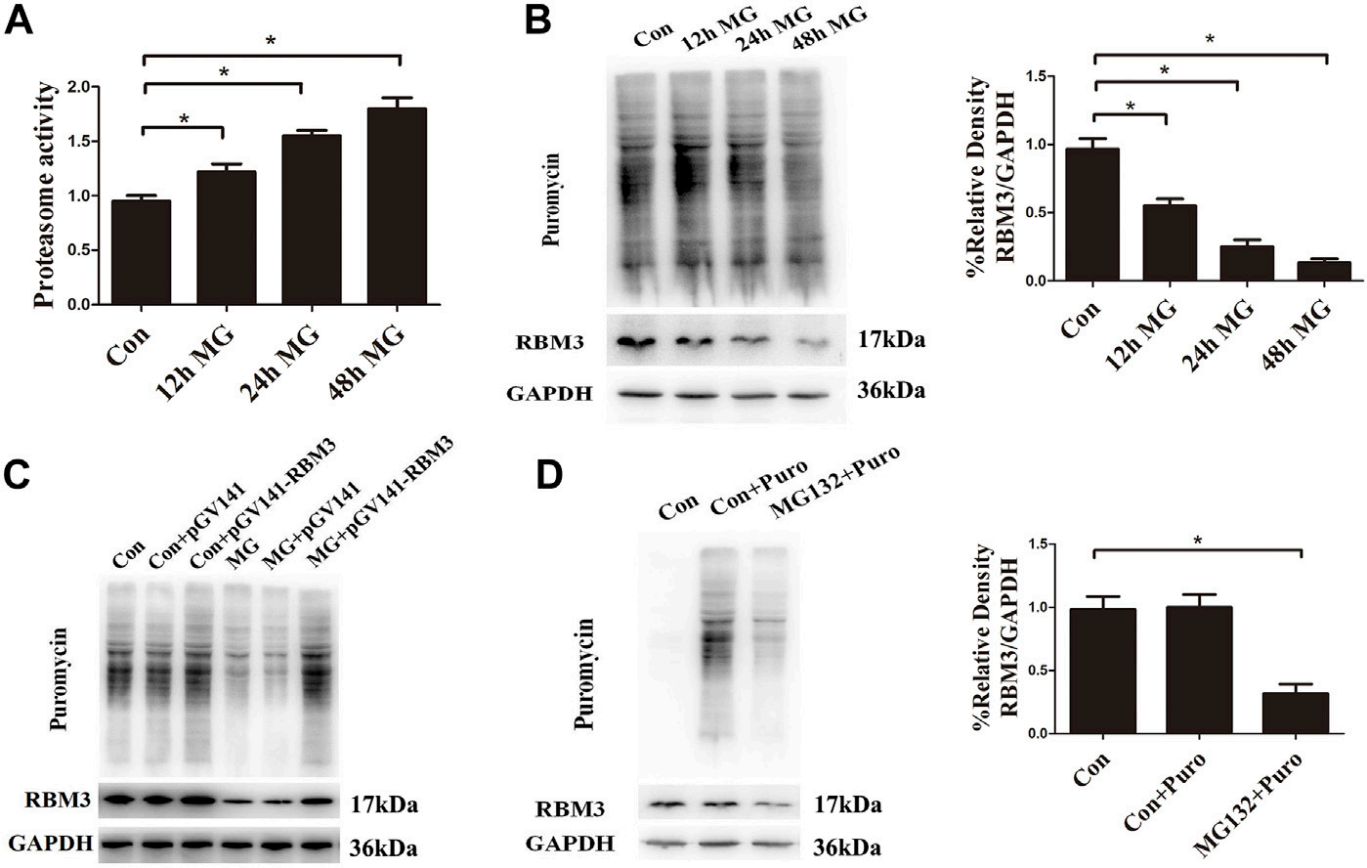
Clinorotation increases proteasome activity and inhibits protein synthesis in HUVECs. **(A)** Activity evaluation for proteasomes in HUVECs exposed to clinorotation for 12, 24, and 48 h. **(B)** Representative Western blot showing the expression of RBM3 and the level of puromycin in HUVECs treated with puromycin for 0.5 h following exposure to clinorotation for 12, 24, and 48 h. **(C)** Representative Western blot showing the level of RBM3 and puromycin in HUVECs transfected with pGV141 or pGV141-RBM3 under clinorotation for 48 h. **(D)** Representative Western blot showing the expression of RBM3 and the level of puromycin in HUVECs treated with or without puromycin for 0.5 h following exposure to MG132 (5 μM) for 12 h. Data shown represented mean ± SD from triplicate experiments. *p < 0.05 vs. the indicated group.

Apart from the activation of chaperones, eukaryotic cells have several other systems to maintain proteostasis, such as ER-associated degradation, autophagy, and impaired protein translation (Su et al., 2016; Baumgartner et al., 2021). Our previous study found that clinorotation-induced autophagy promotes the clearance of ubiquitinated protein aggregates, thus protecting cells against apoptosis (Li et al., 2019). The results in this study indicated that clinorotation for 12, 24, and 48 h increased proteasome activity (Figure 5A). The restraint of the protein synthesis rate after clinorotation for 12, 24, and 48 h was verified by fewer proteins labeled with puromycin using the SUnSET assay (Figure 5B). The cold stress-induced protein RBM3 enhances global levels of protein synthesis at colder temperatures (Dresios et al., 2005). We found that HUVECs exposed to clinorotation for 12, 24, and 48 h displayed lower expression of RBM3 (Figure 5B). To shed light on the role of RBM3 in protein synthesis under simulated microgravity, we used RBM3 overexpression plasmid and found that the slower protein synthesis rate was rescued by RBM3 overexpression under clinorotation (Figure 5C). Moreover, we treated HUVECs with MG132, a proteasome inhibitor that is known to induce intracellular accumulation of misfolded proteins. MG132 significantly reduced the level of RBM3 protein and protein synthesis rate (Figure 5D). These results uncovered the RBM3-mediated restraint of protein synthesis rate under clinorotation. Collectively, coordinated regulation of protein synthesis and degradation along with enhanced autophagy level revealed the proteotoxic stress after clinostat-simulated microgravity.

A few studies have indicated that PINK1 and Parkin are also activated upon accumulation of misfolded proteins in the mitochondrial lumen (Burbulla et al., 2014; Fiesel et al., 2017). Here, we used mitochondrial chaperone Hsp60 as the mtUPR biomarker (Keerthiga et al., 2021; Zhou et al., 2021) and found that the simulated microgravity did not show a much significant effect on the mRNA expression of Hsp60 (Supplementary Figure S3). The result presented here supported that clinorotation activated PINK1 and induced mitophagy independently of mitochondrial-unfolded protein response.

Overall, we concluded that ER stress and UPR due to the imbalance of protein homeostasis upregulated the transfer of Ca^2+^ from the ER to mitochondria through the IP_3_R under clinorotation. The augmented ER-to-mitochondria Ca^2+^ transfer caused a collapse in mitochondrial membrane potential, fission of mitochondria, and mitophagy.

### Clinorotation Activated the NLRP3 Inflammasome by Increasing ROS Levels in HUVECs

**FIGURE 6:**
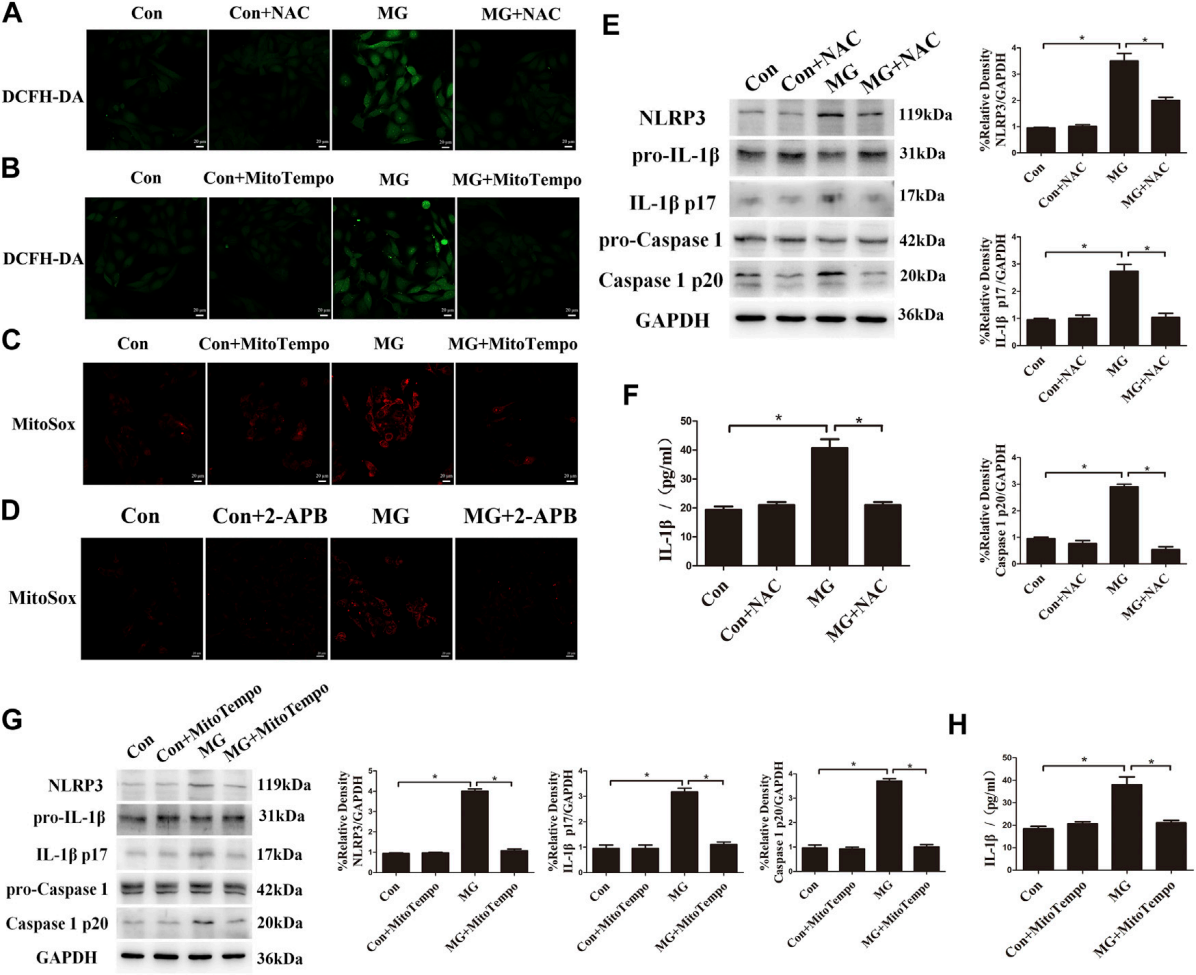
Clinorotation activated the NLRP3 inflammasome by increasing ROS levels in HUVECs. **(A)** Representative microphotographs for the intracellular ROS levels by DCFH-DA staining in HUVECs treated with or without the ROS scavenger NAC (1 µM) under clinorotation for 48 h. **(B)** Representative microphotographs for the intracellular ROS levels by DCFH-DA staining in HUVECs treated with or without mtROS scavenger MitoTEMPO (10 µM) under clinorotation for 48 h. **(C)** Representative microphotographs for MitoSOX staining in HUVECs treated with or without mtROS scavenger MitoTEMPO (10 µM) under clinorotation for 48 h. **(D)** Representative microphotographs for MitoSOX staining in HUVECs treated with or without 2-APB (10 μM) under clinorotation for 48 h. **(E)** Representative Western blot showing the expression of pro-caspase-1, caspase-1 p20, pro-IL-1β, IL-1β p17, and NLRP3 in HUVECs under clinorotation for 48 h with or without ROS scavenger NAC (1 μM). **(F)** Active IL-1β level in cell supernatants following exposure to simulated microgravity for 48 h with or without NAC (1 μM). **(G)** Representative Western blot showing the expression of pro-caspase-1, caspase-1 p20, pro-IL-1β, IL-1β p17, and NLRP3 in HUVECs under clinorotation for 48 h with or without mtROS scavenger MitoTEMPO (10 μM). **(H)** Active IL-1β level in cell supernatants following exposure to simulated microgravity for 48 h with or without MitoTEMPO (10 µM). Data shown represented mean ± SD from triplicate experiments. *p < 0.05 vs. the indicated group. Scale bar: 20 μm.

Mitochondrial ROS are the byproduct of the electron transport chain, and the depolarized and damaged mitochondria lead to the overproduction of ROS (Ma et al., 2018). Based on the strict connection between altered mitochondrial function and mitochondrial ROS, we explored whether ROS production was increased under clinorotation in HUVECs using the DCFH-DA assay. HUVECs under clinorotation exhibited a significant increase in ROS, rescued by NAC, a specific scavenger of ROS (Figure 6A). Mitochondria are an important source of ROS, so HUVECs were incubated in MitoSOX to label mitochondrial superoxide. Treatment with MitoTEMPO, the mitochondria-targeted antioxidant, reduced ROS and mtROS levels under simulated microgravity (Figures 6B,C), signifying mtROS as the main source of ROS under clinorotation. In addition, 2-APB reversed the elevated mtROS level under clinorotation (Figure 6D), suggesting that ER–mitochondria Ca^2+^ transfer was the leading cause of mtROS production.

ROS are well-known promoters of the NLRP3 inflammasome in various cells. Recently, it was reported that simulated microgravity induces NLRP3 inflammasome activation (Jiang et al., 2020). To determine whether mitochondrial ROS are the trigger of the NLRP3 inflammasome in HUVECs after simulated microgravity, the protein levels of the markers of inflammasome such as NLRP3, mature IL-1β, and cleaved caspase 1 were determined. We found increased expression of the inflammatory factors NLRP3, mature IL-1β, and cleaved caspase 1, and the increased release of IL-1β was confirmed by ELISA analysis in HUVECs under clinorotation for 48 h (Figures 6E,F). Treatment with NAC or MitoTEMPO obviously diminished the activation of the NLRP3 inflammasome, indicating that the NLRP3 inflammasome was mtROS-dependent (Figures 6E,G).

**FIGURE 7:**
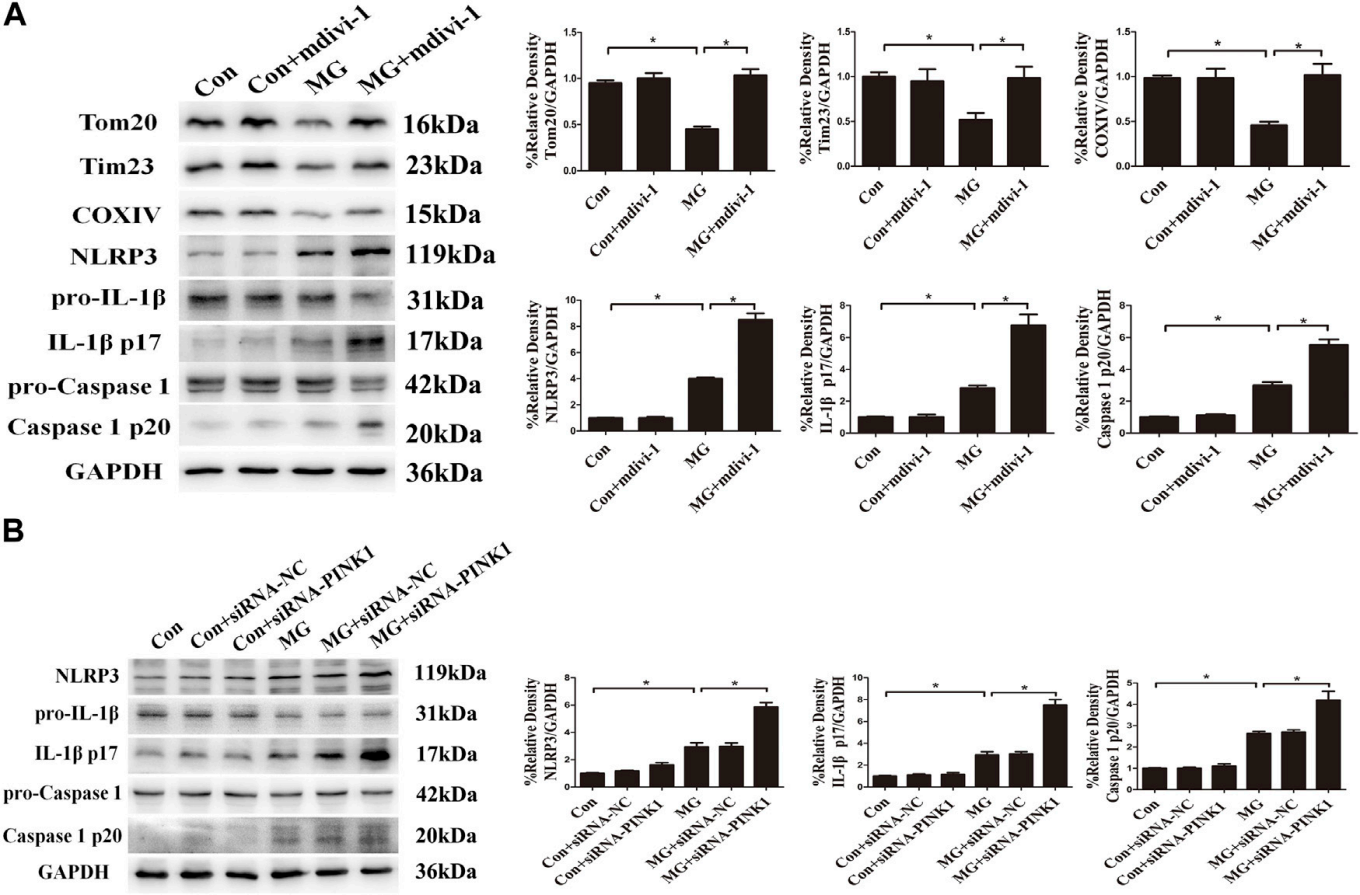
Mitophagy inhibited clinorotation-induced NLRP3 inflammasome activation in HUVECs. **(A)** Representative Western blot showing the expression of Tom20, Tim23, COXIV, pro-caspase-1, caspase-1 p20, pro-IL-1β, IL-1β p17, and NLRP3 in HUVECs under clinorotation for 48 h with or without Drp-1 inhibitor mdivi-1(10 μM). **(B)** Representative Western blot showing the expression of pro-caspase-1, caspase-1 p20, pro-IL-1β, IL-1β p17, and NLRP3 in HUVECs transfected with either siRNA-NC or siRNA-PINK1 in HUVECs exposed to clinorotation for 48 h. Data shown represented mean ± SD from triplicate experiments. *p < 0.05 vs. the indicated group.

Mitochondrial quality control such as mitophagy is protective against oxidative injury in many diseases (Haslip et al., 2015; Zhou et al., 2021). Evidence is accruing that the removal of mitochondria *via* mitophagy prevents apoptosis by reducing mitochondria-derived ROS and subsequent activation of the NLRP3 inflammasome (Chen et al., 2019; Lin et al., 2019). We aimed at assessing whether mitophagy induced by clinorotation ameliorated NLRP3 inflammasome activation *vi*a controlling mtROS production. The mdivi-1 was used as the specific inhibitor of the mitochondrial fission and mitophagy. The effects of mdivi-1 and PINK1 knockdown on the NLRP3 inflammasome were explored using Western blot and ELISA assays. The presence of mdivi-1 under clinorotation made the expression of NLRP3, IL-1β p17, and caspase 1 p20 higher than that of the MG group (Figure 7A). Similarly, PINK1 knockdown exacerbated the activation of the NLRP3 inflammasome (Figure 7B).

To demonstrate that the inhibition of NLRP3 inflammasome by mitophagy is linked to mitochondrial ROS production, we investigated the effect of MitoTEMPO treatment on the NLRP3 inflammasome. As shown in Supplementary Figure S4A, the mitochondrial ROS level was markedly increased when PINK1 was knocked down by siRNA under simulated microgravity. As expected, the increased mtROS level and activation of the NLRP3 inflammasome induced by clinorotation were abolished by the presence of MitoTEMPO (Supplementary Figure S4B). Taken together, these results suggested that mitophagy inhibited NLRP3 inflammasome activation by reducing mitochondria-derived ROS production.

### Mitophagy Inhibited Endothelial Hyperpermeability and Negatively Regulates Cellular Migration in HUVECs Under Clinorotation

**FIGURE 8:**
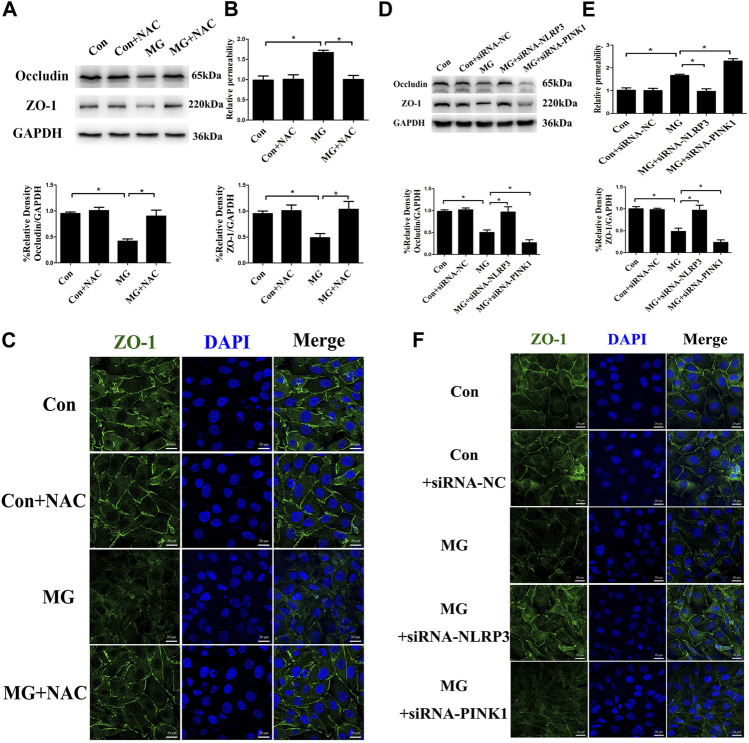
Mitophagy inhibited NLRP3 inflammasome activation–induced endothelial tight junction disruption and increased endothelial permeability in HUVECs. **(A)** Representative Western blot showing the expression of endothelial junction proteins occludin and ZO-1 in HUVECs under clinorotation for 48 h with or without ROS scavenger NAC (1 µM). **(B)** Relative dextran permeability of HUVECs exposed to clinorotation for 48 h with or without NAC (1 µM). **(C)** Representative fluorescence images showing ZO-1 (green) in HUVECs exposed to clinorotation for 48 h with or without NAC (1 µM). **(D)** Representative Western blot showing the expression of occludin and ZO-1 in HUVECs transfected with siRNA-NC, siRNA-NLRP3, or siRNA-PINK1 under clinorotation for 48 h. **(E)** Relative dextran permeability of HUVECs transfected with siRNA-NC, siRNA-NLRP3, or siRNA-PINK1 under clinorotation for 48 h. **(F)** Representative fluorescence images showing ZO-1 (green) in HUVECs transfected with siRNA-NC, siRNA-NLRP3, or siRNA-PINK1 under clinorotation for 48 h. Data shown represented mean ± SD from triplicate experiments. *p < 0.05 vs. the indicated group. Scale bar: 20 μm.

The vascular endothelium barrier contributes to vascular homeostasis. Endothelium alteration by the reduced level of tight-junction proteins induces endothelial hyperpermeability, which is the pathogenesis of many human diseases. Many stimuli induce changes in endothelial permeability *via* ROS and the NLRP3 inflammasome (Boini et al., 2017; Zhang et al., 2019; Zhou et al., 2019). To investigate whether ROS and the NLRP3 inflammasome induced by clinorotation cause the disassembly of tight-junction proteins, we examined the expression of the representative tight-junction proteins ZO-1 and occludin. As shown in Figure 8A, simulated microgravity decreased the expression of ZO-1 and occludin. Moreover, dextran flux significantly increased in HUVECs under clinorotation for 48 h (Figure 8B). Consistently, our confocal analysis showed that clinorotation markedly decreased the expression of the tight-junction protein ZO-1 on endothelial cell monolayers (Figure 8C). This clinorotation-induced increase in EC permeability was markedly reduced in the presence of NAC or NLRP3 siRNA transfection (Figure 8D). Next, we detected the role of mitophagy in EC permeability under clinorotation, given that mitophagy ameliorated ROS production and NLRP3 inflammation activation. It was found that PINK1 knockdown aggravated the inter-endothelial junction disruption (Figures 8D–F). Therefore, our results indicated that mitophagy attenuated endothelial hyperpermeability induced by clinorotation.

**FIGURE 9:**
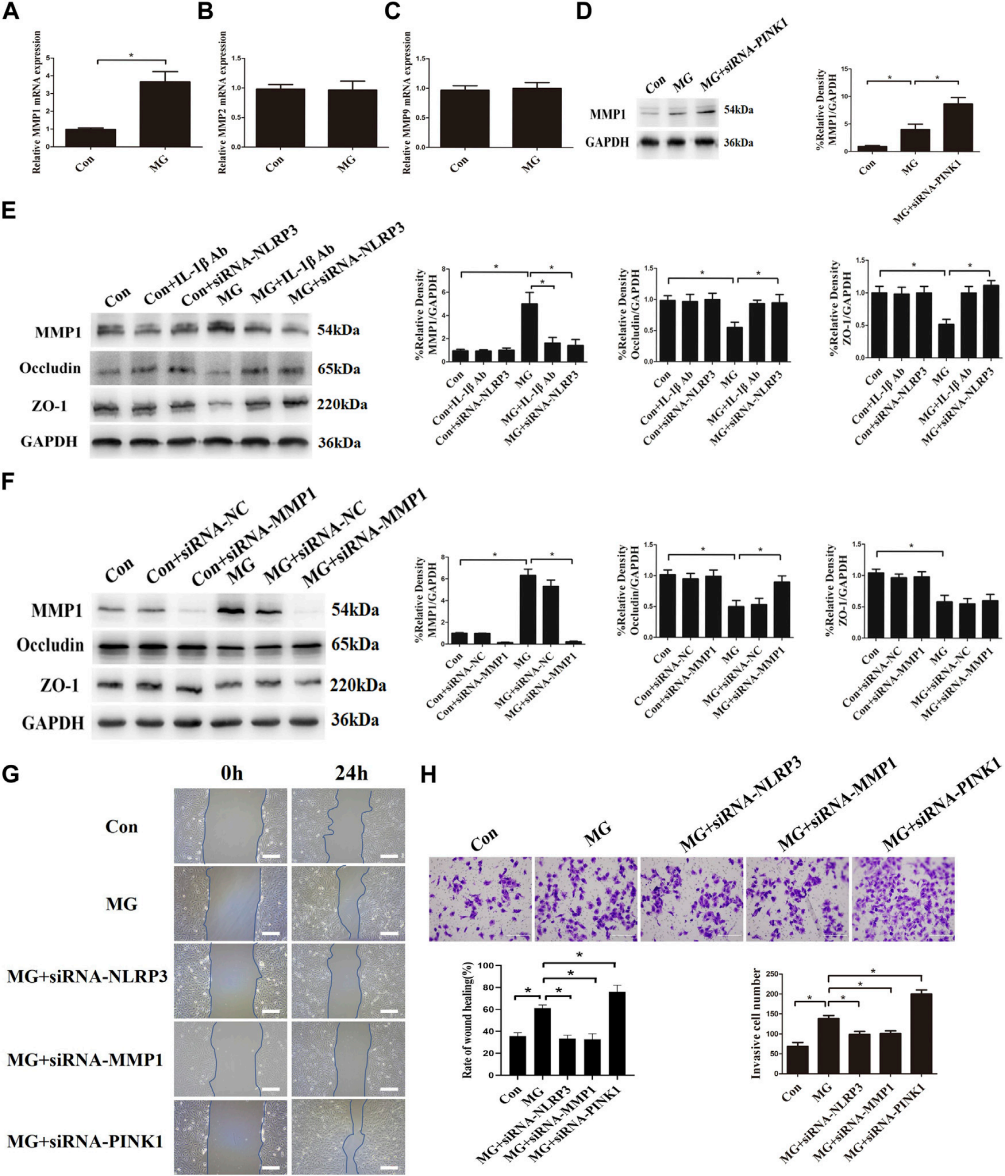
Clinorotation induced mitophagy and NLRP3 inflammasome activation influenced endothelial tight junction and cell migration partly by inducing the expression of MMP1 in HUVECs. **(A,B,C)** mRNA expression of MMP1 **(A)**, MMP2 **(B),** and MMP9 **(C)** in HUVECs under clinorotation for 48 h. **(D)** Representative Western blot showing the expression of MMP1 in HUVECs transfected with siRNA-PINK1 under clinorotation for 48 h. **(E)** Representative Western blot showing the expression of MMP1, occludin, and ZO-1 in HUVECs treated with neutralizing IL-1β antibody (1 μg/ml) or transfected with siRNA-PINK1 under clinorotation for 48 h. **(F)** Representative Western blot showing the expression of MMP1, occludin, and ZO-1 in HUVECs transfected with siRNA-NC or siRNA-MMP1 in HUVECs exposed to clinorotation for 48 h. **(G)** Transwell migration assay was performed to evaluate the migration capacity of HUVECs transfected with siRNA-NLRP3, siRNA-MMP1, or siRNA-PINK1 under clinorotation for 48 h. **(H)** A wound healing assay was performed to evaluate the migration capacity of HUVECs. Data shown represented mean ± SD from triplicate experiments. *p < 0.05 vs. the indicated group. Scale bar: 200 μm.

The integrity of endothelial cells is regulated by MMPs, which are destructive to adhesion and tight-junction structures by degrading tight-junction proteins (Pan et al., 2021). To determine the influence of the MMP family on the permeability of HUVECs, the expressions of MMP1, MMP2, and MMP9 were explored. Clinorotation increased the mRNA and protein expression of MMP1 but had no effect on mRNA levels of MMP2 and MMP9 (Figures 9A–C). In addition, HUVECs treated with PINK1 siRNA had significantly increased MMP1 compared to that of the control group (Figure 9D), indicating that mitophagy inhibited MMP1 expression. Pro-inflammatory cytokine IL-1β is known to promote MMP expression (Kaden et al., 2003; Chen et al., 2018). We hypothesized that mitophagy could suppress MMP1 expression by inhibiting the activation of the NLRP3 inflammasome and IL-1β. To verify our conjecture, HUVECs were transfected with NLRP3 siRNA or subjected to IL-1β neutralizing antibody under clinorotation. As shown in Figure 9E, the presence of IL-1β neutralizing antibody or NLRP3 gene silencing prevented the changes in protein levels of MMP1, ZO-1, and occludin induced by clinorotation, suggesting that increased IL-1β release after NLRP3 inflammasome formation contributed to increased expression of MMP1 and disassembly of tight-junction proteins ZO-1 and occludin. To provide direct evidence for the involvement of MMP1 in the disrupted endothelial tight junction, we examined the effect of MMP1 siRNA on the protein level of ZO-1 and occludin. It is interesting to note that MMP1 knockdown mitigated the decrease of occludin expression under clinorotation, whereas it failed to rescue downregulated ZO-1 expression (Figure 9F). These data suggested that the effects of IL-1β on tight-junction proteins ZO-1 and occludin may be mediated by other unknown ways apart from MMP1.

In addition to endothelium barrier function, cell migration is a key aspect of endothelial cells which could be regulated by MMP1 (Li et al., 2018b). Transwell migration assay and wound healing assay were performed to assess the cellular ability of migration in HUVECs transfected with siRNA-NLRP3, siRNA-MMP1, or siRNA-PINK1 under simulated microgravity. Genetic deletion of MMP1 or NLRP3 reversed the increased cellular migration, whereas PINK1 knockdown under clinorotation enhanced the cellular migration compared to the MG group (Figures 9G,H). These results confirmed that mitophagy negatively regulated cellular migration *via* the NLRP3-MMP1 pathway.

## Discussion

**FIGURE 10:**
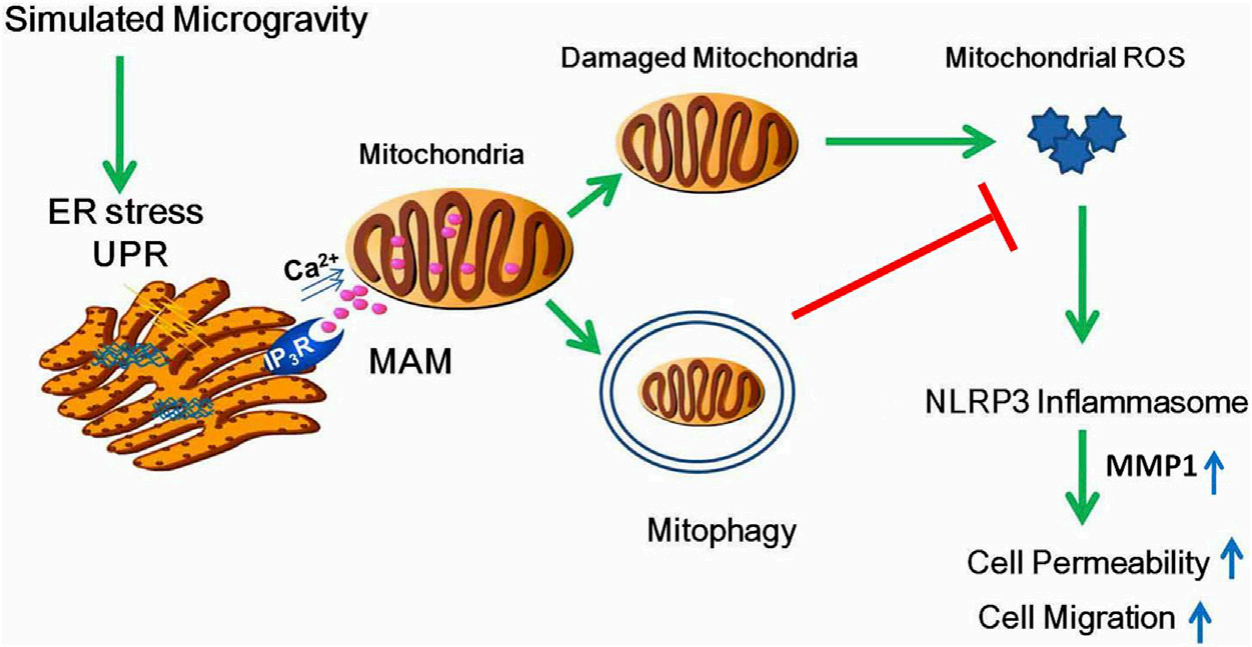
Schematic diagram depicting the signaling pathway of mitophagy induced by clinostat-simulated microgravity and the role of mitophagy in endothelial functional changes. Proteotoxic stress activated by microgravity leads to ER stress and subsequently Ca^2+^ transfer from the ER to mitochondria through IP_3_R, resulting in mitochondria Ca^2+^ overload, increased fission, mitochondrial membrane potential collapse, and mitophagy. Mitophagy inhibits permeability and cell migration by ameliorating ROS production which induces NLRP3 inflammasome activation.

In the current study, we used 2-D clinostat as the simulated microgravity model and demonstrated for the first time that proteostasis failure–induced ER stress promotes Ca^2+^ transfer to mitochondria, which results in mitochondria fission and mitophagy. In addition, we also demonstrated that enhanced elimination of damaged mitochondria alleviates endothelial hyperpermeability and cellular migration ability through the inhibition of mtROS-induced NLRP3 inflammasome activation (Figure 10).

Vascular endothelial cell is a mechanical-sensitive cell type, with possible functional changes ranging from enhanced cellular migration or ER stress or increased autophagy level (Li et al., 2018a) to elevated apoptosis rate (Li et al., 2019) under microgravity. Microgravity as a mechanical stressor has been reported to interfere with mitochondrial function. The downregulation of genes involved in oxidative phosphorylation under microgravity contributes to mitochondrial dysfunction and pro-oxidative environment (Versari et al., 2013). Simulated microgravity leads to lower intracellular ATP levels, overproduction of ROS, and decreased mitochondrial mass in human Hodgkin’s lymphoma cells (Jeong et al., 2018). Even though several studies have shown that mitochondrial dysfunction occurs under microgravity (da Silveira et al., 2020; Locatelli et al., 2020), yet there is a lack of clear understanding of the mechanism of how mitochondrial dysfunction and mitophagy take place and how mitochondrial dysfunctions contribute to endothelial functional changes. The PINK1/Parkin pathway is the best-characterized pathway of mitophagy. PINK1, normally imported into the mitochondrion and continuously degraded at the inner mitochondrial membrane, accumulates in its full‐length form on the outer mitochondrial membrane of damaged mitochondria, which exhibits loss of membrane potential. The phosphorylation of outer mitochondrial membrane proteins by PINK1 leads to the recruitment and activation of Parkin from the cytosol to the mitochondria to ubiquitinate various OMM substrates such as fusion protein Mfn2. The ubiquitination of Parkin substrates on the OMM facilitates the recruitment of autophagic adaptors such as p62 which serve as anchors for the autophagosomal membrane, thus mediating autophagic clearance of damaged mitochondria (Stotland and Gottlieb, 2015). In the present study, we confirmed clinorotation-induced mitophagy *via* the PINK1/Parkin pathway, as shown by colocalization of Tom20 with LC3 or MitoTracker with LysoTracker and by accumulation of PINK1, Parkin, and p62 in mitochondrial extracts. Of note, a recent study shows that the upregulation of BNIP3 may play a crucial role in mitophagy induced by simulated microgravity for 4 d and 10 d using a rotating wall vessel (Locatelli et al., 2020). The different data in simulated microgravity conditions may be due to different lengths of simulated microgravity and the method of how HUVECs are exposed to a weightless environment.

Fission of reticulate mitochondria into smaller fragments is a precondition for mitophagy. The key regulator of this process is Drp1 which is in concert with other pro-fission proteins such as Fis1, Mff, and MiD49. Mitochondrial depolarization recruits Drp1, leading to mitochondrial fragmentation. In addition, ubiquitination and proteasomal degradation of Mfn2 prevent the targeted mitochondria from fusion (Buhlman et al., 2014). We showed that HUVECs under clinorotation exhibited a decrease in the Mfn2 protein level, an increase in the Drp1 protein level, mitochondrial fission, and mitochondrial membrane potential loss. To our knowledge, the results provide the first experimental evidence which demonstrated that exposure to 2-D clinostat-simulated microgravity for 48 h could decrease mitochondrial membrane potential, promote fission, and facilitate PINK1/Parkin-mediated mitophagy in HUVECs.

Multiple known mechanisms contribute to the relationship between ER stress and mitophagy. The ER stress response can modulate mitophagy *via* the activation of IRE1α/ATF6–XBP1, which promoted the transcription of E3 ligase MARCH5 to facilitate the ubiquitination and degradation of MFN2 (Wang et al., 2021). XBP1, a transcription factor activated upon ER stress, was shown to control mitophagy by transactivating PINK1 in neurons (El Manaa et al., 2021). Given the fact that the PINK1 protein level was elevated under microgravity, whether PINK1 was transactivated needs further research. In addition, the ER as the main intracellular store of Ca^2+^ is physically and physiologically interconnected with mitochondria *via* Ca^2+^ fluxes. ER stress has been previously shown to increase Ca^2+^ transfer from ER to mitochondria. IP_3_R–Grp75–VDAC complex, located at the mitochondria–ER contact sites, serves as the Ca^2+^ channel (Hedskog et al., 2013). The fact that ER stress occurs in HUVECs after exposure to clinorotation (Li et al., 2019) led us to speculate that ER stress could impact mitochondrial health and lead to accumulation of mitochondrial Ca^2+^. Here, we demonstrated that Ca^2+^ transfer from the ER to mitochondria through IP_3_R increased in HUVECs under clinorotation. An overload in mitochondrial Ca^2+^ caused by Ca^2+^ release from the ER has been implicated in mitochondrial fragmentation and mitochondrial membrane potential collapse. Homocysteine induced a decreased mitochondrial membrane potential in endothelial cells due to accumulation of mitochondrial Ca^2+^, depending on the upregulated expression of the IP_3_R–Grp75–VDAC complex (Chen et al., 2021). Hyperglycemia promotes MAM formation, accumulation of mitochondrial Ca^2+^, mitochondrial depolarization, and fission in cardiomyocytes (Wu et al., 2019). In line with these reports, we found that IP_3_R-dependent Ca^2+^ release from ER into mitochondria resulted in mitochondrial depolarization and fission. Although the regulatory role of IP_3_R in mitophagy and calcium homeostasis is confirmed by many studies (Arruda et al., 2014; Moulis et al., 2019), there is a lack of direct evidence supporting that ER-to-mitochondria Ca^2+^ transfer through IP_3_R leads to mitophagy. Our study offers evidence supporting that excessive Ca^2+^ transport from the ER to mitochondria contributes to mitophagy under clinorotation in HUVECs.

Even though mounting evidence indicates ER stress is a key contributor to the dysfunction of various cells under microgravity, yet there is a lack of clear understanding of the mechanism of how ER stress takes place. Research confirms that excessive accumulation of unfolded or misfolded proteins inside the ER lumen activates ER stress, which concurrently results in the activation of a signaling network known as UPR (Ron and Walter, 2007). Microgravity has been previously reported to trigger protein aggregation and protein misfolding in human endothelial cells, which is reflected by upregulation of Hsp70 and formation of perinuclear clusters of actins (Cazzaniga et al., 2019). Proteostasis loss also occurs in *C. elegans* in response to spaceflight (Honda et al., 2012). Moreover, exposure to simulated microgravity alters ribosome assembly and mitochondrial and cytosolic translation pathways, which are related to proteostasis failure in both human and murine models (da Silveira et al., 2020). Consistent with these findings, our study found the proteotoxic stress and activation of the major protein quality control mechanisms in HUVECs under microgravity, as evidenced by increased expression of heat shock protein, increased level of autophagy and ubiquitinated protein (Li et al., 2019), and decreased protein synthesis and increased proteasome activity. However, a previous report indicates that global ubiquitination and protein degradation are not influenced by microgravity in primary cardiac cells, although simulated microgravity causes global downregulation of ribosomal proteins and protein synthesis rate (Feger et al., 2016). We suggest that different types of cells may exhibit different ways of maintenance of proteome homeostasis under microgravity. In addition, we first showed the increased expression of a secreted protein chaperone clusterin under microgravity, which is found to be enhanced after proteotoxic stress (Loison et al., 2006). It is interesting to note that decreased expression of the cold-induced protein and RNA-binding protein RBM3 contributed to protein synthesis inhibition. RBM3 is found to facilitate global protein synthesis by binding to 60S ribosomal subunits to enhance translation directly or by changing the number of microRNAs (Dresios et al., 2005). Future studies are required to further understand the link between the downregulation of RBM3 and decreased protein synthesis under microgravity. Taken together, proteostasis failure drives mitochondria fragmentation, dysfunction, and mitophagy by inducing ER stress and subsequently heavy Ca^2+^ influx to mitochondria.

Previous studies have proven that excessive Ca^2+^ transfer from the ER to mitochondria due to proteotoxic stress promotes mtROS generation (Joy et al., 2021). We found a similar increase in ROS and mtROS, which were reversed by NAC and MitoTEMPO, respectively. There is much research evidence on the ROS generation under microgravity so far. Human Hodgkin’s lymphoma cells exposed to 3D-clinostat–simulated microgravity exhibit increased ROS production (Jeong et al., 2018). Simulated microgravity induces ROS generation in human promyelocytic leukemia cells, leading to DNA damage and apoptosis (Singh et al., 2021). The oxidative stress in HUVEC under clinostat prompts us to explore the link between ROS and endothelial cell function. The NLRP3 inflammasome is a protein complex activated by damage-associated molecular patterns in response to cellular stress. NLRP3 activation leads to the assembly of NLRP3, ASC, and caspase-1, which in turn leads to proteolytic activation of the pro-IL-1β and pro-IL-18. This promotes inflammation and induces inflammatory, pyroptotic cell death through the release of cytokines, such as IL-1β and IL-18, and other molecules (Nanda et al., 2021). A recent study has found that the NLRP3 inflammasome contributes to endothelial inflammation and apoptosis associated with microgravity (Jiang et al., 2020). The secretion of IL-1α and IL-1β was increased in HUVECs on board the ISS under microgravity (Versari et al., 2013). In muscle tissues of mice, the inflammatory markers such as IFN-γ, IL-1, and tumor TNF gene are upregulated by spaceflight, accounting for muscle wasting (da Silveira et al., 2020). Here, we found that clinorotation for 48 h promoted the release of IL-1β due to NLRP3 inflammasome activation. However, it is worth noting that space microgravity suppresses the secretion of pro-inflammatory cytokines such as IL-8 and IL-1β in EA. hy926 ECs cultured on board the SJ-10 Recoverable Scientific Satellite. Different cell types and different time frames of microgravity exposure may explain the contradiction.

Several mechanisms underlying inflammasome activation have been reported, including lysosome rupture, K^+^ channel gating, and ROS. In order to verify the role of ROS in the activation of the NLRP3 inflammasome, we used ROS scavenger NAC and mtROS scavenger MitoTEMPO and found that the protein levels of NLRP3, mature IL-1β, and cleaved caspase 1 decreased in the presence of NAC and MitoTEMPO, confirming that ROS was involved in NLRP3 inflammasome activation. In addition to ROS, cytoplasmic mtDNA and synthesis of new mtDNA have been shown to be required for NLRP3 activation (Coll et al., 2018). Whether the release of mtDNA to cytosol due to mitochondrial damage and mitophagy under simulated microgravity contributes to NLRP3 activation remains to be further elucidated. It is widely appreciated that activating mitophagy could reduce NLRP3 inflammasome activation. Mitophagy defect in ECs causes leakage of mitochondrial DNA, which results in NLRP3 inflammasome activation (Wu et al., 2021). The removal of mitochondria *via* mitophagy prevents apoptosis of renal tubular epithelial cells by reducing mitochondria-derived ROS and subsequent activation of the NLRP3 inflammasome (Lin et al., 2019). In arthritis rat models, resveratrol inhibits the activation of NLRP3 inflammasomes by inducing the PINK1/Parkin-dependent mitophagy (Fan et al., 2021). In our study, we found mitophagy defect by PINK1 knockdown or in the presence of mdivi-1–enhanced ROS production and NLRP3 inflammasome activation. The mtROS scavenger MitoTEMPO could reverse NLRP3 inflammasome activation induced by mitophagy inhibition. Our results confirmed that mitophagy could alleviate an elevated NLRP3 inflammasome level by reducing ROS production in HUVECs under simulated microgravity.

Vascular endothelium as a barrier controls the passage of materials and contributes to vascular homeostasis. The integrity of inter-endothelial tight junctions plays a central role in the vascular endothelial barrier. The main protein constituents of tight junctions in endothelium include occludin, claudin-5, and ZO-1. ZO-1 is a membrane localizing scaffold protein that is critical for the connection between tight-junction proteins. Occludin, binding to ZO-1, localizes to bicellular tight junctions. Many stimuli can act on endothelial permeability such as a high-fat diet and LPS. The permeability of the blood–brain barrier increases in rats exposed to simulated microgravity by tail suspending, and 3-D clinorotation downregulates the protein levels of ZO-1 and occludin in human brain microvascular endothelial cells (Yan et al., 2021). It has been well-established that the activation of the NLRP3 inflammasome is an important initiating mechanism leading to endothelial dysfunction. TMAO-induced disassembly of tight-junction protein ZO-1 and the increase in EC permeability have been shown to be prevented by NLRP3 knockdown (Boini et al., 2017). LPS induces endothelial layer dysfunction by activating the NLRP3 inflammasome in microvascular endothelial cells (Zhou et al., 2019). In this study, the expressions of occludin and ZO-1 were downregulated, and FITC-dextran permeability was increased in HUVECs under clinorotation. The hyperpermeability could be mitigated by NLRP3 knockdown and NAC treatment and could be aggravated by mitophagy inhibition. To our knowledge, our findings demonstrate for the first time that clinostat-induced endothelial barrier defects, which are associated with NLRP3 inflammasome–dependent tight junction disruption, could be compromised by mitophagy. Next, we examined how NLRP3 inflammasome activation induced endothelial barrier disruption. The roles of MMPs, involved in degrading the extracellular matrix, are well-documented in cell migration, invasion, and angiogenesis. MMP1 is reported to degrade occludin and claudin, breaching the blood–brain barrier (Wu et al., 2015). High MMP expression is closely related to inflammatory conditions. IL-1β promotes the expression of MMP1, MMP3, and MMP13 in stenotic calcific aortic valves (Kaden et al., 2003) and promotes the secretion of MMP1 in mesenchymal stem cells (Chen et al., 2018). In this report, we have shown that MMP1, but not MMP2 and MMP9, was upregulated which could be reversed by IL-1β neutralizing antibody or NLRP3 knockdown, while blocking mitophagy enhanced the high expression of MMP1, suggesting that the upregulation of MMP1 is due to NLRP3 inflammasome activation–induced secretion of IL-1β. It should be noted that knockdown of MMP1 blocked the degradation of occludin but not ZO-1, whereas treatment of IL-1β neutralizing antibody blocked the degradation of both occludin and ZO-1. This result, which is consistent with the previous report which shows that MMP1 degrades occludin but has no effect on ZO-1 (Wu et al., 2015), indicates that other downstream mediators of IL-1β could contribute to mediating ZO-1 degradation other than MMP1. For example, NLRP3 inflammasome activation is reported to facilitate the release of cytokine HMGB1, leading to a decrease of ZO-1 (Zhou et al., 2019), but whether HMGB1 mediates ZO-1 degradation under microgravity remains to be elucidated in the future. In addition, MMP1 is reported to be necessary for the migration of human bone marrow-derived mesenchymal stem cells (Chen et al., 2018) and plays an important role in the migration of vascular smooth muscle cells (Austin et al., 2013). Given that the cellular migration of HUVECs was enhanced by simulated microgravity (Li et al., 2018a), we evaluated the role of MMP1, NLRP3 inflammasome, and mitophagy in cell migration ability in HUVECs under clinorotation. We found that cellular migration was significantly attenuated by NLRP3 knockdown or MMP1 knockdown, whereas the migration ability was enhanced when mitophagy was abolished, implying mitophagy inhibits endothelial migration *via* inhibiting the activation of the NLRP3 inflammasome and the high expression of MMP1.

In summary, we unravel that enhanced PINK1-dependent mitophagy in HUVECs after exposure to simulated microgravity inhibits the activation of the NLRP3 inflammasome, hyperpermeability of the endothelial cell monolayer, and enhanced cell migration under clinorotation. Then, we delineate the pathway that leads to mitophagy under microgravity. ER stress and UPR under clinorotation induced by proteotoxic stress cause exaggerated ER–mitochondria Ca^2+^ transfer through IP_3_R, leading to Ca^2+^ accumulation in mitochondria, which results in the collapse of mitochondrial membrane potential, mitochondrial fission, and mitophagy. This study deepens the understanding of the mechanism of cardiovascular dysfunction under microgravity and may help provide potential therapeutic actions to protect the cardiovascular system.

## Data Availability

The datasets presented in this study can be found in online repositories. The names of the repository/repositories and accession number(s) can be found in the article/Supplementary Material.

## Abbreviations

ASC, apoptosis-associated speck-like protein containing CARD; BNIP3, Bcl-2/adenovirus E1B-19 kDa-interacting protein 3; Drp1, dynamin-related protein 1; FUNDC1, FUN14 domain-containing 1; HMGB1, high-mobility group box 1; HSF1, heat shock factor 1; Hsp70, heat shock protein 70; PMSF, phenylmethylsulfonyl fluoride; PBS, phosphate-buffered saline; HUVECs, human umbilical vein endothelial cells; IRE1, inositol-requiring element 1; NLRP3, NOD-like receptor pyrin domain containing 3; LC3, microtubule-associated protein 1 light chain 3; MG, microgravity; MAMs, mitochondria-associated membranes; FITC, fluorescein isothiocyanate; PVDF, polyvinylidene difluoride; ELISA, enzyme-linked immunosorbent assay; LIR, microtubule-associated protein 1 light chain 3 interacting region; HRP, horseradish peroxidase; SDS-PAGE, sodium dodecyl sulfate-polyacrylamide gel electrophoresis; Tom20, translocase of the outer mitochondrial membrane 20; Tim23, translocase of the inner mitochondrial membrane 23; ER, endoplasmic reticulum; ECL, enhanced chemiluminescence; ROS, reactive oxygen species; mdivi-1, mitochondrial division inhibitor-1; TEM, transmission electron microscopy; Mfn2, mitofusin 2; NIX, NIP3-like protein X; NAC, N-acetylcysteine; MitoSOX, mitochondrially targeted superoxide indicator; MMPs, matrix metalloproteinases; mtROS, mitochondrial reactive oxygen species; mtUPR, mitochondrial unfolded protein response; OMM, outer mitochondrial membrane; PINK1, PTEN-induced putative kinase 1; RBM3, RNA-binding protein 3; UPR, unfolded protein response; IP3R, inositol trisphosphate receptor; VDAC, voltage-dependent anion channel; Grp75, 75 kDa glucose-regulated protein; COXIV, cytochrome c oxidase IV; ZO-1, zonula occludens-1; TMAO, trimethylamine N-oxide; LPS, lipopolysaccharide.
