# Supplementary material for: PINK1-Dependent Mitophagy Reduced Endothelial Hyperpermeability and Cell Migration Capacity Under Simulated Microgravity
Source: Front Cell Dev Biol. 2022 Jul 7;10:896014. doi: 10.3389/fcell.2022.896014 (PMC9300855; doi:10.3389/fcell.2022.896014)
Supplement: Supplementary file 1 [file Presentation1.pdf]

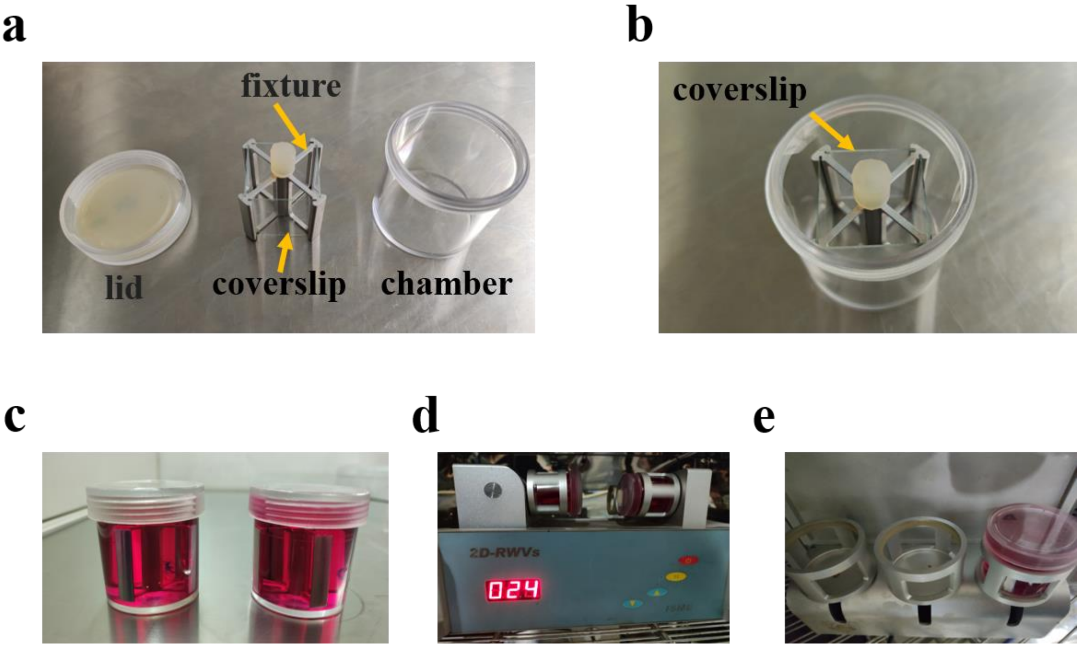

Figure S1. Representative pictures of the 2-D clinostat facility. (a) The lid, coverslip, fixture and chamber. (b) Representative picture of chamber with fixture and coverslips. (c) Chambers before rotation which were filled with complete cell culture medium after removing air bubbles. (d) Microgravity group using clinorotation around the horizontal axis with the rotational speed of 24r/min. (e) Control group using clinorotation around the vertical axis with the same speed as the microgravity group.

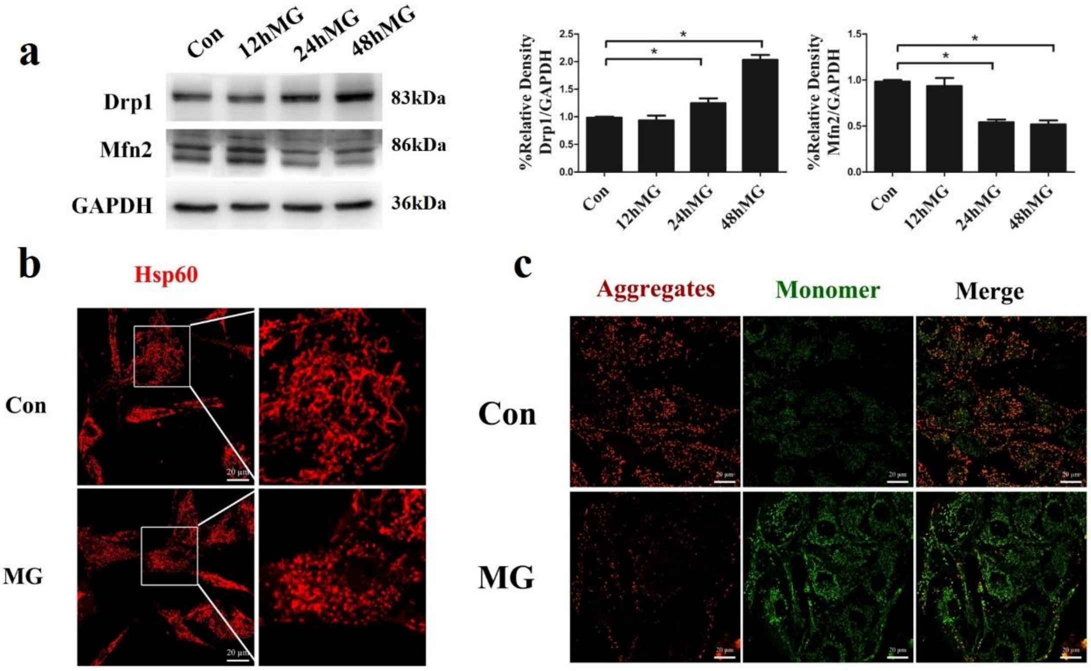

Figure S2. Clinorotation induced mitochondrial fission and decreased mitochondrial membrane potential in HUVECs. (a) Representative western blot showing expression of pro-fission protein Drp1 and pro-fusion protein Mfn2 in HUVECs exposed to simulated microgravity for 12h, 24h and 48h. GAPDH was probed

as a protein loading control. (b) Immunofluorescence staining for Hsp60 (red) to indicate the morphology of mitochondria in HUVECs under clinorotation for 48h. (c) JC-1 assay was used to examine mitochondrial membrane potential in HUVECs exposed to clinorotation for 48h. Data shown represented mean ± SD from triplicate experiments. *P < 0.05 vs the control. Scale: 20μm.

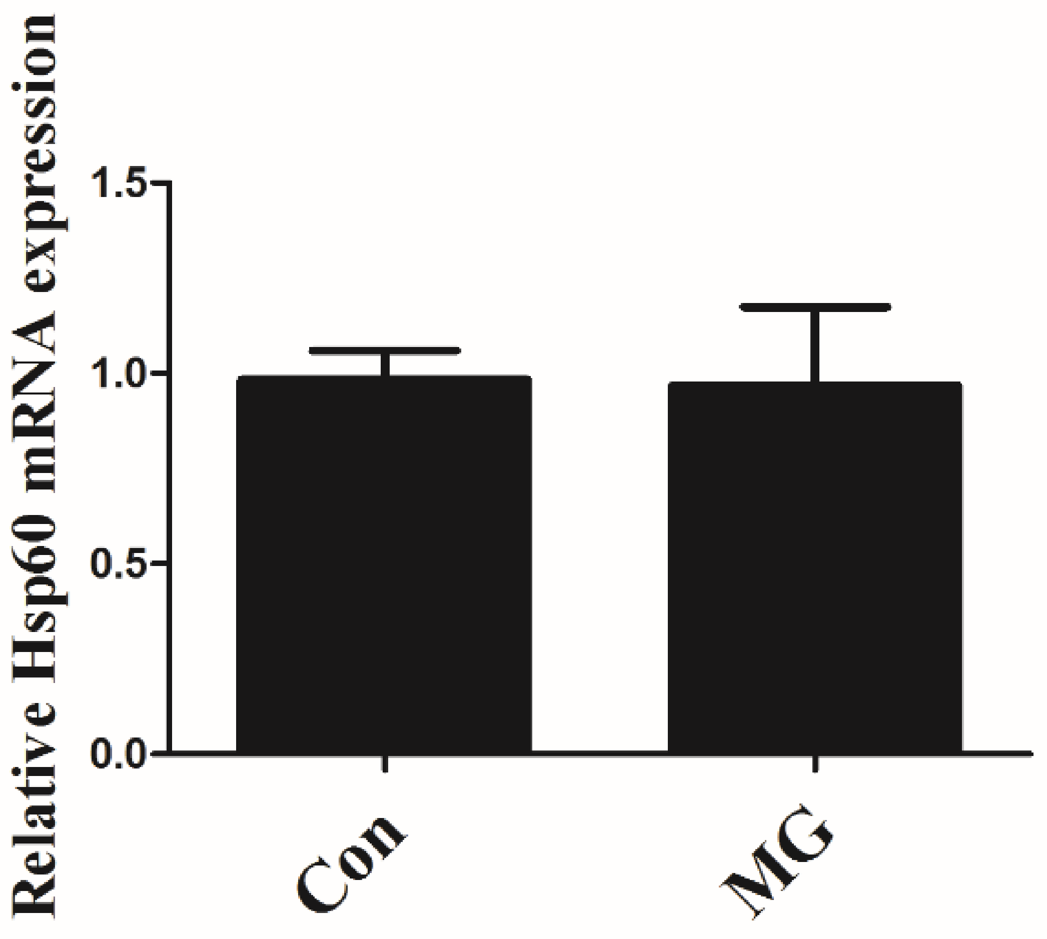

Figure S3. The mRNA expression of mtUPR marker Hsp60 in HUVECs under clinorotation for 48h.

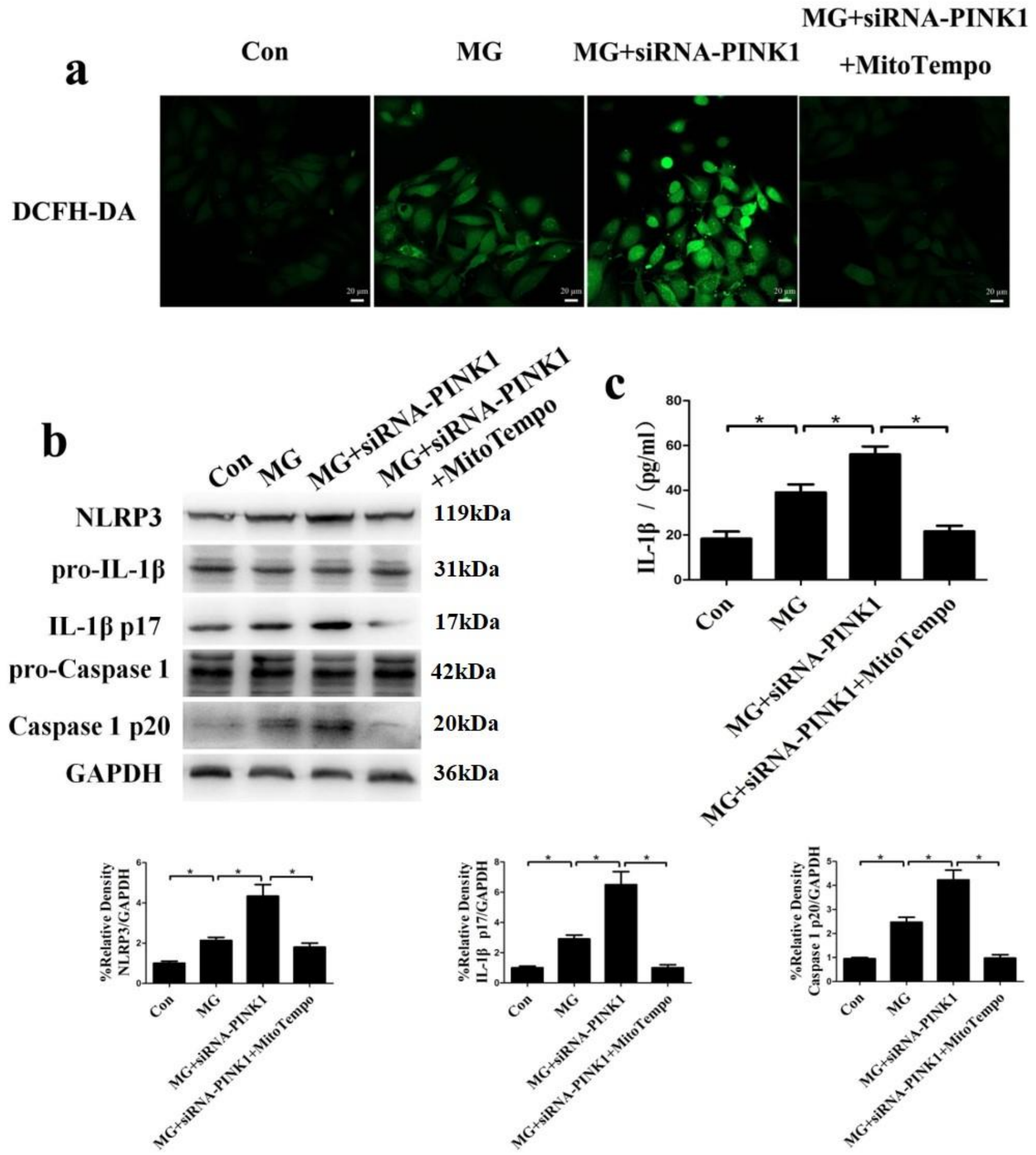

Figure S4. Mitophagy inhibited clinorotation-induced NLRP3 inflammasome activation in HUVECs by inhibiting production of ROS. (a) Representative microphotographs for the intracellular ROS levels by DCFH-DA staining in HUVECs transfected with siRNA-PINK1 with or without mtROS scavenger MitoTempo (10μM) under clinorotation for 48h. (b) Representative western blot showing expression of pro-Caspase 1, Caspase 1 p20, pro-IL-1β, IL-1β p17 and NLRP3 in HUVECs transfected with siRNA-PINK1 with or without mtROS scavenger MitoTempo (10μM) under clinorotation for 48h. (c) Active IL-1β levels in cell supernatants. Data shown represented mean ± SD from triplicate experiments. *$P < 0.05$ vs the indicated group. Scale bar: 20μm.
